# A Genuine Stannylone with a Monoatomic Two‐Coordinate Tin(0) Atom Supported by a Bis(silylene) Ligand

**DOI:** 10.1002/anie.202114073

**Published:** 2021-12-01

**Authors:** Jian Xu, Chenshu Dai, Shenglai Yao, Jun Zhu, Matthias Driess

**Affiliations:** ^1^ Department of Chemistry: Metalorganics and Inorganic Materials Technische Universität Berlin Strasse des 17. Juni 115, Sekr. C2 10623 Berlin Germany; ^2^ State Key Laboratory of Physical Chemistry of Solid Surface and Collaborative Innovation Center of Chemistry for Energy Materials (iChEM) and College of Chemistry and Chemical Engineering Xiamen University 361005 Xiamen People's Republic of China

**Keywords:** Carbonyl Iron Complexes, Chelate ligands, Stannylone, Silylene, Tin

## Abstract

The monoatomic zero‐valent tin complex (stannylone) {[Si^II^(Xant)Si^II^]Sn^0^} **5** stabilized by a bis(silylene)xanthene ligand, [Si^II^(Xant)Si^II^=PhC(NtBu)_2_Si(Xant)Si(NtBu)_2_CPh], and its bis‐tetracarbonyliron complex {[Si^II^(Xant)Si^II^]Sn^0^[Fe(CO)_4_]_2_} **4** are reported. The stannylone **5** bearing a two‐coordinate zero‐valent tin atom is synthesized by reduction of the precursor **4** with potassium graphite. Compound **4** results from the Sn^II^ halide precursor {[Si^II^(Xant)Si^II^]Sn^II^Cl}Cl **2** or {[Si^II^(Xant)Si^II^]SnBr_2_} **3** through reductive salt‐metathesis reaction with K_2_Fe(CO)_4_. According to density functional theory (DFT) calculations, the highest occupied molecular orbital (HOMO) and HOMO‐1 of **5** correspond to a π‐type lone pair with delocalization into both adjacent vacant orbitals of the Si^II^ atoms and a σ‐type lone pair at the Sn^0^ center, respectively, indicating genuine stannylone character.

## Graphical abstract

The bis(*N*‐heterocyclic silylene)‐stabilized stannylone with a two‐coordinate Sn^0^ atom and its bis‐Fe(CO)_4_ complex have been synthesized and structurally characterized by NMR spectroscopy and X‐ray crystallography. Their bonding situations have been uncovered by density functional theory calculations, indicating a genuine stannylone character with two lone pairs of electrons at the tin atom.

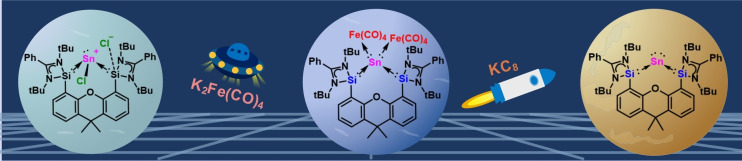

Monoatomic zero‐valent metal complexes are very well established in transition‐metal chemistry.^[1]^ In contrast, related compounds of the main‐group elements are far less explored. Efforts to realize isolable monoatomic zero‐valent Group 14 underwent a boost when Frenking and co‐workers reinterpreted the donor–acceptor interactions in carbodiphosphorane based on quantum chemical analysis, showing that the compound has a diphosphine carbon(0) complex character.^[2]^ As a consequence, the existence of a new series of complexes with the general form L:→E^0^←:L (L=σ‐donor, E=C, Si, Ge, Sn, Pb; Scheme 1) was predicted theoretically.^[3]^ In addition, the term “ylidone” (E=C: carbone; Si, silylone; Ge, germylone; Sn, stannylone; Pb, plumbylone) was given to this type of compounds.^[3b]^ Owing to the zero‐valent nature of the central atoms, which possess four valence electrons as two lone pairs, the synthesis of such isolable species has attracted much attention in the last decade. Utilizing different strong donor ligand systems, several isolable carbones, silylones, and germylones have been realized experimentally.[^4^, ^5^, ^6^, ^7^] Moving down to the heaviest Group 14 elements, tin and lead, however, the synthesis of such complexes becomes rather difficult due to their intrinsic lability which readily leads to decomposition of the products as free ligands and elemental tin or lead. To date, a genuine two‐coordinate stannylone and plumbylone featuring two lone pairs of electrons at the central Sn^0^ and Pb^0^ atom remain unknown.

**Scheme 1:**
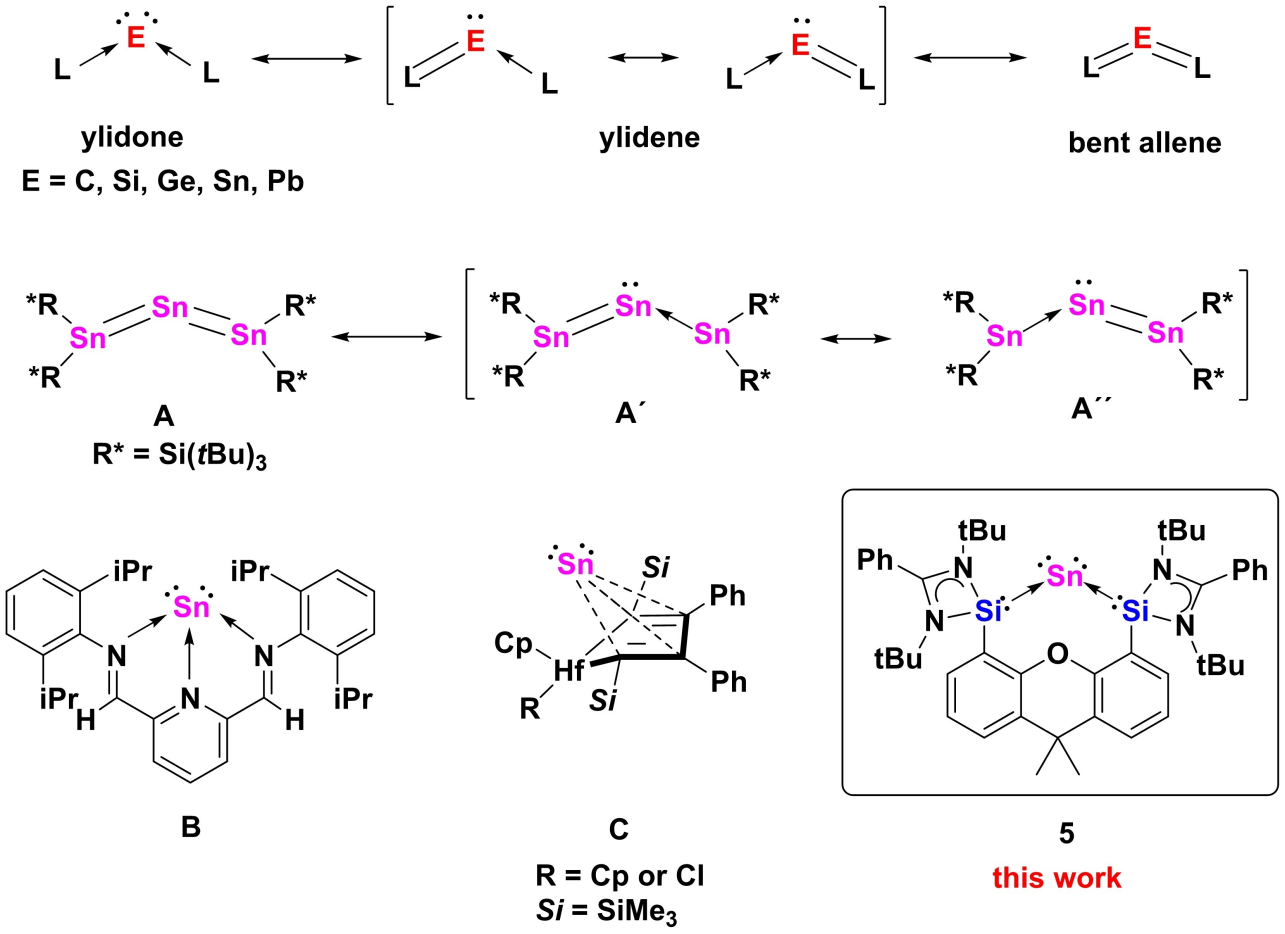
Top: General form of ylidones, ylidenes and bent‐allene resonance structures. Mid: Tristannaallene **A** and its resonance structures **A′** and **A′′**. Bottom: Examples of the Sn^0^ complexes **B**, **C**, and **5** of this work.

By combining valence bond (VB) theory and maximum probability domain (MPD) approaches, Turek and co‐workers suggested that heavy group 14 zero‐valent complexes should be described as a resonating combination of ylidone and ylidene structures with a minor contribution of the bent allene form (Scheme 1).^[8]^ Remarkably, the tristannaallene **A** ^[9]^ (Scheme 1), reported by Wiberg and co‐workers in 1999, adopts a bent allene structure. According to the ^119^Sn NMR spectrum and theoretical calculations, **A** exhibits low‐valent character with only one lone pair at the central tin atom. It was thus also described as a stannylene adduct of a distannavinylidene (**A′** or **A′′**). Employing a tridentate diiminopyridine, Flock and co‐workers have obtained complex **B** (Scheme 1) containing a tin atom in the formal oxidation state of zero, which may also be described as a Sn^II^ complex due to the presence of a redox non‐innocent ligand.^[10]^ Notably, Saito and co‐workers documented recently the *η* ^4^‐butadiene Sn^0^ complex **C** ^[11]^ (Scheme 1) featuring some transition‐metal‐like behavior for a p‐block metal. In recent years, we were interested in the chemistry of monoatomic zero‐valent Group 14 complexes and succeeded in isolating several silylones and germylones with chelating bis(NHC) (NHC=*N*‐heterocyclic carbene) and bis(NHSi) (NHSi=*N*‐heterocyclic silylene) chelating ligands, respectively.[^6c^, ^6d^, ^6e^, ^7d^, ^7e^, ^7f^] Herein, we report the synthesis of the genuine stannylone **5** with a monoatomic two‐coordinate Sn^0^ atom supported by a bis(NHSi)xanthene ligand (Scheme 1) and its bis‐Fe(CO)_4_ complex **4**. The structures of **2**, **3**, **4** and **5** were confirmed by X‐ray crystallographic studies.^[12]^

Starting from the chelating bis(NHSi)xanthene Si^II^(Xant)Si^II^ ligand **1**,^[13]^ the reaction with one molar equivalent of SnCl_2_(dioxane) and SnBr_2_(dioxane) in Et_2_O at room temperature resulted in a yellow precipitate of the bis(NHSi)‐Sn^II^ halide complexes {[Si^II^(Xant)Si^II^]SnCl}Cl **2** and {[Si^II^(Xant)Si^II^]SnBr_2_} **3**, which were isolated in 85 % and 72 % yields, respectively (Scheme 2). The ^29^Si NMR spectra of **2** and **3** show a singlet at *δ*=29.4 ppm (^1^ *J*_Si, Sn_=1334 Hz) and 30.3 ppm (^1^ *J*_Si, Sn_=1350 Hz) with ^119^Sn‐satellites, respectively, downfield‐shifted with respect to that of **1** (*δ*=17.3 ppm). The ^119^Sn NMR spectra show a singlet at *δ*=−348.1 ppm for **2** and *δ*=−392.0 ppm for **3**.

**Scheme 2:**
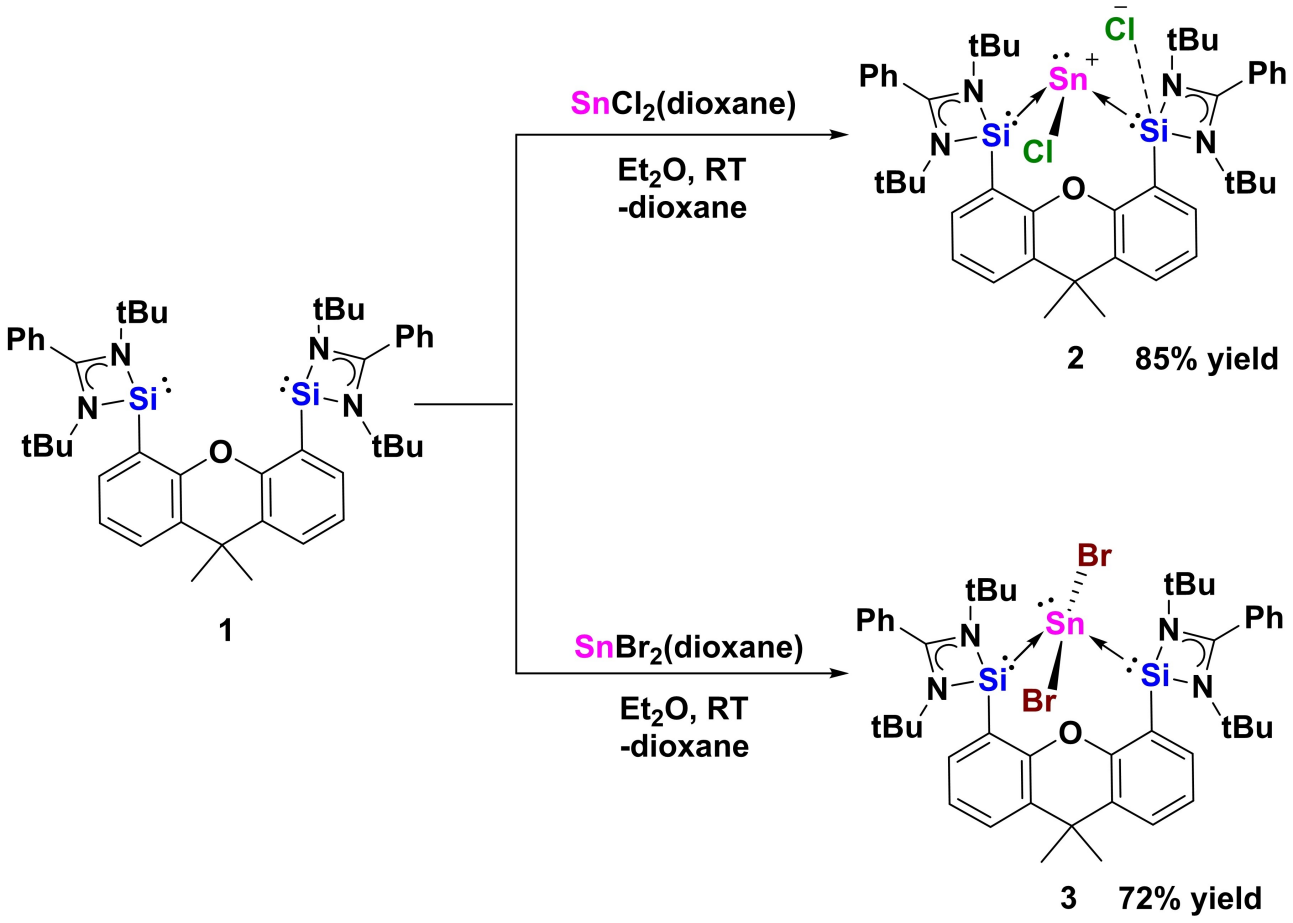
Synthesis of the bis(NHSi)‐Sn^II^ halide complexes **2** and **3** from bis(NHSi) **1**.

The molecular structure of **2** established by X‐ray diffraction (XRD) reveals a bis(NHSi) supported Sn^II^ center bonded to a chlorine atom (Cl1) and a stereoactive lone pair, adopting a trigonal‐pyramidal coordination geometry (Figure 1 left). The remaining chloride anion Cl2 shows a weak interaction with one of the silicon atoms (Si2⋅⋅⋅Cl2: 2.52(2) Å) akin to the situation in previously reported analogous chlorogermyliumylidene chloride.^[7e]^ In the case of **3** (Figure 1 middle), the central Sn^II^ atom is four‐coordinate. Featuring also a stereoactive lone pair, the Sn^II^ center adopts a see‐saw geometry with two bromine atoms located in the axial positions (Br1‐Sn1‐Br2: 165.79(13)°).

**Figure 1:**
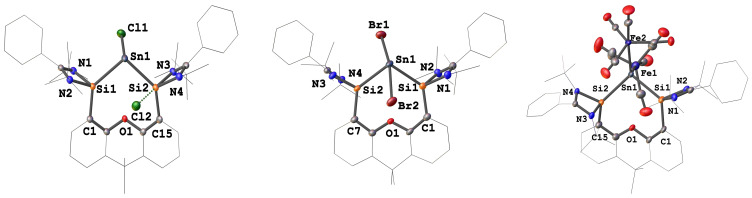
Molecular structures of **2**, **3** and **4**.^[12]^ Thermal ellipsoids are drawn at the 50 % probability level. H atoms and solvent molecules are omitted for clarity.

Following the successful approach for the synthesis of silylones^[6]^ and germylones,^[7]^ we attempted to synthesize a stannylone through reduction of **2** and **3** with KC_8_ or Na(C_10_H_8_) but failed. Presumably, the thus‐formed zero‐valent tin complex supported by the strong σ‐donating bis(NHSi) ligand is too sensitive under the harsh reducing conditions. Considering our previous success of synthesizing the bis(NHSi)pyridine germylone iron carbonyl complex {[SiNSi]Ge^0^→Fe(CO)_4_},^[14]^ the introduction of Fe(CO)_4_ as a Lewis acid may increases the stability of the desired stannylone. Compound **2** was thus mixed with 1.2 molar equivalents of Collman's reagent,^[15]^ [K_2_Fe(CO)_4_], in THF and stirred overnight at room temperature, leading to the desired bis‐Fe(CO)_4_ stannylone complex **4** in 42 % yields (Scheme 3). The mechanism of **4** is still unknown, however, we reasoned that reduction of **2** and **3** with K_2_Fe(CO)_4_ firstly affords the 1 : 1 Fe(CO)_4_ complex **4′** similar to {[SiNSi]Ge^0^→Fe(CO)_4_}^[14]^ as an intermediate (Scheme 3). Compound **4′** could be labile with respect to liberation of “free” Fe(CO)_4_ which, in turn, reacts with intact **4′** to afford the bis‐Fe(CO)_4_ complex **4** as final product.

**Scheme 3:**
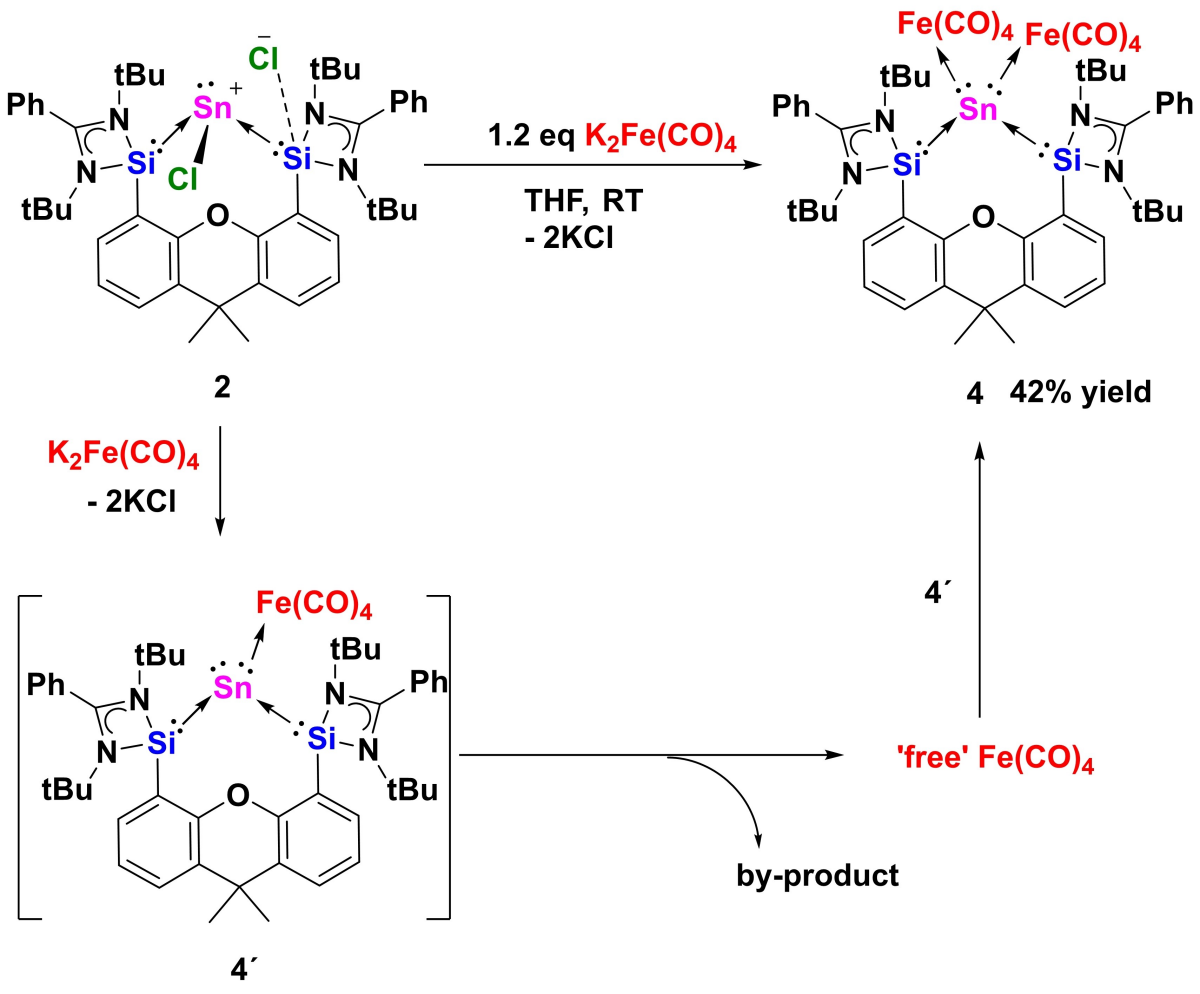
Formation of diiron carbonyl stannylone complex **4** via **4′** from **2**.

The ^29^Si NMR spectrum of **4** shows a singlet at *δ*=27.0 (^1^ *J*_Si, Sn_=1312 Hz) ppm, while the ^119^Sn NMR spectrum exhibits a singlet at *δ*=7.3 ppm. The molecular structure of **4** displays a bis(NHSi)‐supported central Sn atom coordinated to two independent Fe(CO)_4_ moieties in a distorted tetrahedral geometry (Figure 1 right). The Sn−Si distances in **4** (2.68(11) and 2.70(12) Å) are comparable to those values in **2** and **3** (2.62–2.71 Å), respectively. The Sn−Fe distances of 2.60(7) Å and 2.65(9) Å are considerably longer than those observed in related Sn^II^→Fe(CO)_4_ complexes (ca. 2.40–2.50 Å).^[16]^ The IR stretching vibrations of CO in **4** appear at 1962, 1881 and 1834 cm^−1^, which are significantly red‐shifted compared to the values of a carbonyl *N*‐heterocyclic stannylene iron complex (2055, 1973, and 1953 cm^−1^).^[17]^ This indicates that the Sn^0^ center of **4** is a stronger electron donor than the stannylene‐Sn^II^ atoms, despite that the capability of the latter stannylene‐Sn^II^ atoms as π‐acceptors should not be ignored.

To gain an insight into the electronic structure of **4**, we performed density functional theory (DFT) calculations with the Gaussian 16 (Revision A.03) program.^[18]^ All structures were optimized at the PEB0‐D3BJ/Def2‐SVP∼ma‐TZVPlevel of theory in the gas phase due to the smallest relative mean deviation (RD) of structural parameters in comparison with the metric data from X‐ray structure analyses. No imaginary frequency was obtained at the same level, confirming a local minima. The full sets of calculated geometries and energies are given in Supporting Information.

The principal interacting orbital (PIO) analysis^[19]^ evaluates the strength of orbital interactions via PIO‐based bond index (PBI). As a result, we found four dominating σ‐type interactions between the Sn and other bonded atoms (Figure 2). The second and third PIO pairs correspond to two dative bonds between Sn and two Si atoms with close‐to‐one PBI values, 0.91 and 0.88, respectively. Specifically, the Sn atom utilizes its *5p* orbitals to form two Sn−Si bonds with contributions of 1.31 e/1.35 e from Si and 0.69 e/0.65 e from Sn. For the first PIO pair, the lone pair electrons on the *p*‐orbital of Sn atom are decreased to 0.88 e, caused by σ‐donation to *d*‐orbitals of Fe. In addition, the other lone pair electrons on the Sn atom donate to the *d*‐orbitals of the Fe atoms in the fourth PIO pair. In other words, the Sn atom in **4** accepts electrons from the two adjacent donor Si^II^ atoms and donates electrons to Fe, forming four dative bonds with the Si and Fe atoms. Furthermore, these four donating interactions between Sn and four adjacent atoms (two Si and two Fe) are consistent with the results of the natural adaptive orbital (NAdO) analysis^[20]^ (Figure S28). Specifically, four σ‐type NAdOs are determined with close‐to‐one eigenvalues, 0.826, 0.806, 0.703 and 0.660, respectively.

**Figure 2:**
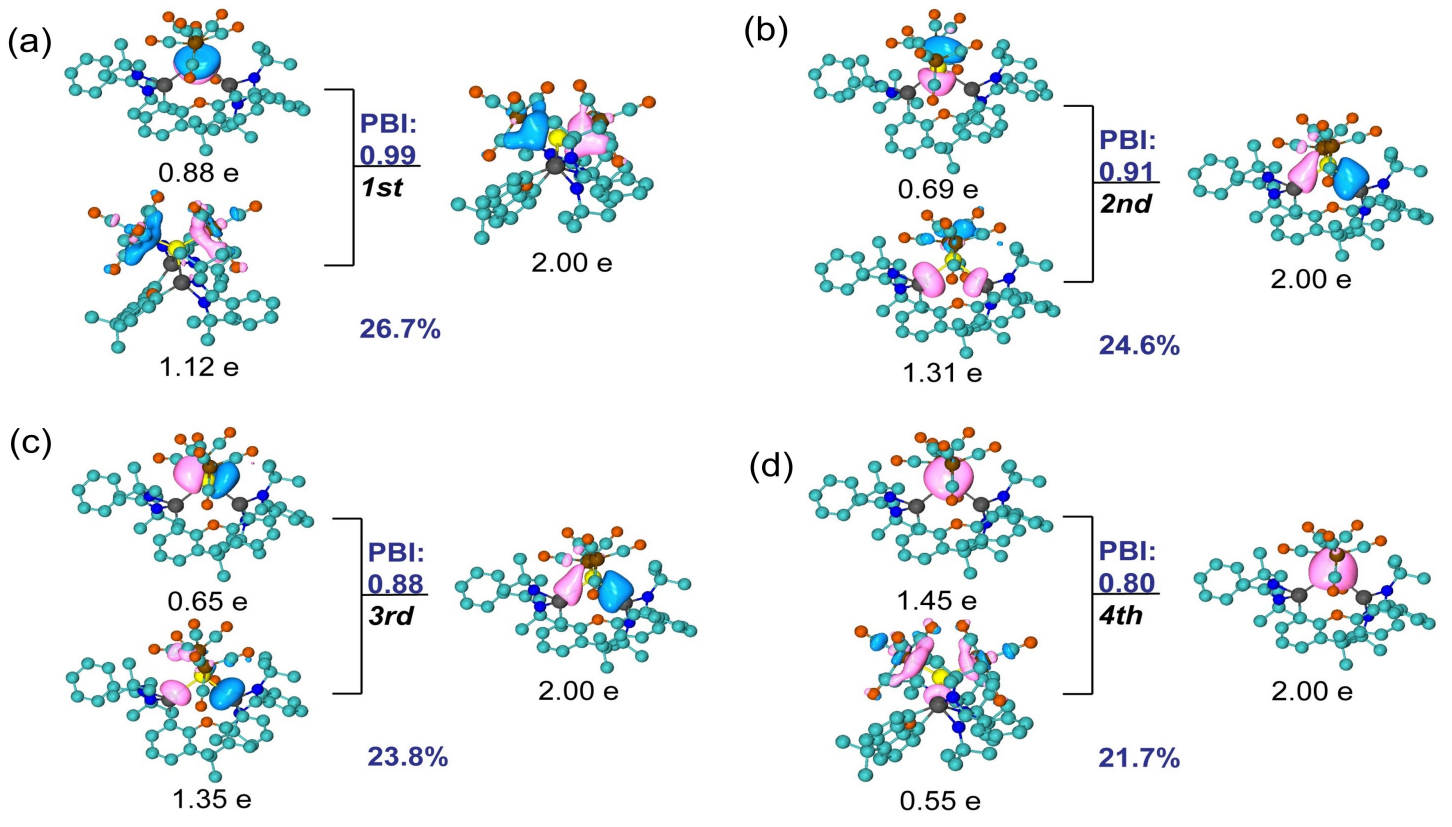
PIO analysis on the bonding modes of Sn−Si and Sn−Fe in compound **4**. The compound **4** is decomposed to two moieties, the Sn atom and the rest. Hydrogen atoms in 3D structures are omitted for clarity. The total PBI value of two fragments is 3.68. The isosurface 0.050 au is plotted.

Since compound **4** can be considered as a carbonyl stannylone iron complex with a tin center in the formal zero oxidation state, it could be a more suitable precursor to generate the desired stannylone than **2** and **3**. In order to eliminate the [Fe(CO)_4_] moieties as [Fe(CO)_4_]^2−^ leaving groups, **4** was allowed to react with KC_8_ in a molar ratio of 1 : 4.1 in THF and stirred at room temperature for six hours (Scheme 4). To our delight, the desired reductive elimination of [Fe(CO)_4_] succeeds and leads to the formation of the dark blue stannylone **5** which could be isolated in 56 % yields from the reaction mixture. The dark blue color of **5** fades immediately when exposed to air, indicating its high sensibility towards oxygen and moisture. In the solid state, stannylone **5** can be stored under N_2_ atmosphere. However, it does gradually decompose into elemental tin and free bis(NHSi)xanthene ligand **1** in solutions (NMR). The ^29^Si NMR spectrum of **5** shows a singlet at *δ*=50.6 ppm with ^119^Sn‐satellites (^1^ *J*_Si,Sn_=1244 Hz), downfield‐shifted relative to **4** (*δ*=27.0 ppm) and **1** (*δ*=17.3 ppm). The ^119^Sn NMR spectrum exhibits a singlet at *δ*=−1147.2 ppm, dramatically highfield‐shifted with respect to that of **4** (*δ*=7.3 ppm). The enormous upfield shift observed for **5** is confirmed by Gauge‐Independent Atomic Orbital (GIAO) calculations (*δ*_cal_.=−1325.5 ppm, Supporting Information). We reasoned that the remarkable shielding of the ^119^Sn nucleus in **5** is caused by the two “free” lone‐pairs of electrons at the Sn^0^ center. In addition, **5** can be converted back to **4** by reaction with Fe_2_(CO)_9_ in THF under release of CO (Scheme 4).

**Scheme 4:**
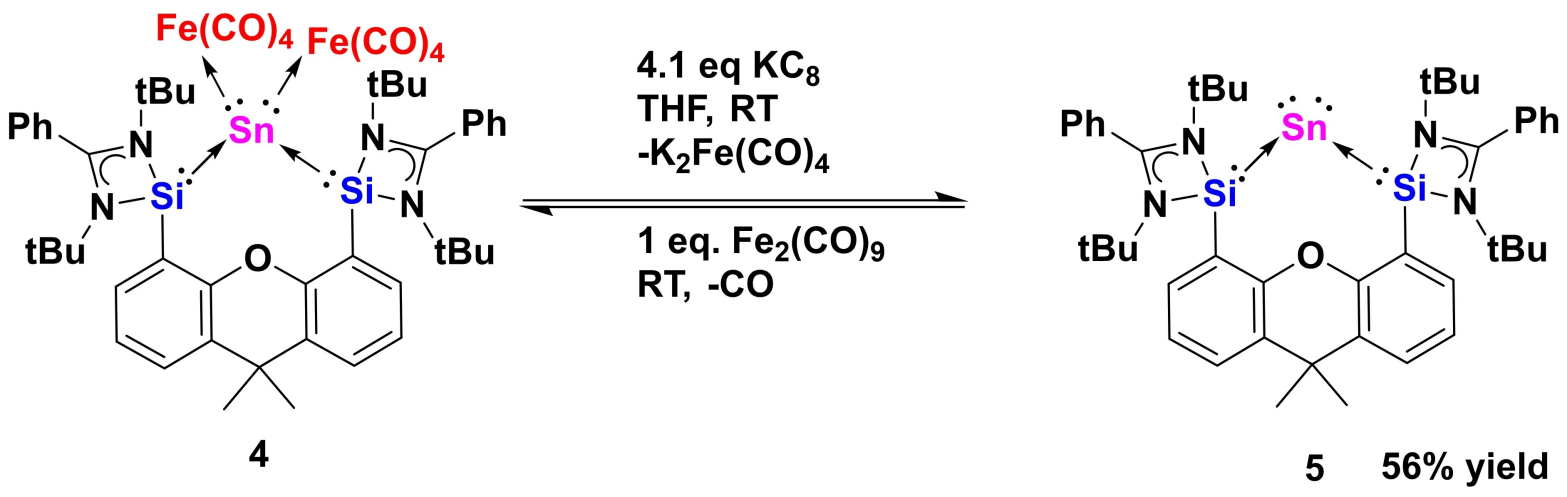
Conversion of complex **4** to stannylone **5** with KC_8_ and its reversed reaction with Fe_2_(CO)_9_.

Compound **5** crystallizes in the monoclinic space group *P*2_1_/*c* with two independent molecules in the asymmetric unit (Figure 3). The central Sn^0^ atoms are coordinated by the two Si^II^ atoms of the bis(NHSi) donor with the Si1−Sn1−Si2 angles of 99.34(10)° and 100.10(9)°, respectively. The Si−Sn distances, ranging from 2.51(3) to 2.54(3) Å, are significantly shorter than those in **4** (2.68(11) and 2.70(12) Å) and intermediate between a Sn−Si double [(2.42(14) Å)]^[21]^ and Si−Sn single bond lengths of 2.60 Å.^[22]^

**Figure 3:**
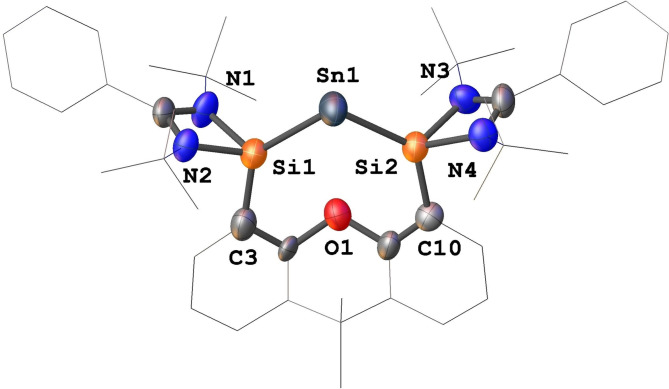
Molecular structure of **5**.^[12]^ The asymmetric unit contains two independent molecules, only one is depicted. Thermal ellipsoids are drawn at the 50 % probability level. H atoms are omitted for clarity.

Also, we performed a PIO analysis for **5** and found two σ‐types and a π‐type interaction between the Sn and two Si atoms (Figure 4b). The first and third PIO pairs correspond to σ‐type dative interaction with close‐to‐one PBI values, 0.92 and 0.81, respectively, similar to those of **4**. In addition, the π‐type delocalization between Sn and the two Si atoms could be formed by the lone pair electrons of a *5p* orbital on the Sn atom interacting with the adjacent vacant *3p* orbitals of the Si atoms. The larger fuzzy bond order than 1.0 (approximately 1.3) for Sn−Si is also the result of a π‐type delocalization. Furthermore, these three primary interactions between Sn and the two Si atoms are supported by NAdO analysis (Figure S30), where two σ‐type and a π‐type NAdOs were located with close‐to‐one Eigenvalues, 0.866, 0.825 and 0.661, respectively. As shown in Figure 4a, the HOMO‐1 and HOMO, respectively, correspond to a σ‐lone pair and a π‐lone pair on the Sn^0^ atom. The latter is significantly delocalized into the adjacent Si atoms. In addition to the ylidone structure of **5**, we thus propose the relevance of the two ylidene resonance structures **5′** and **5′′** (Scheme 5). The UV/Vis spectrum of **5** recorded in toluene displays an intense absorption at 674 nm with the absorptivity *ϵ*_max_/(M^−1^ cm^−1^) of 3088 (Figure S22). According to time‐dependent DFT (TD‐DFT) calculations,^[23]^ the observed peak (λ_ex_=674 nm, λ_TD‐DFT_=672 nm) is mainly assigned to the S_1_ state (Table S14), which corresponds to the π‐π* excitation from HOMO to LUMO. Notably, the band is more red‐shifted than that of the silicon (silylone, 569 nm)^[6d]^ and germanium (germylone, 596 nm)^[7a]^ homologues.

**Figure 4:**
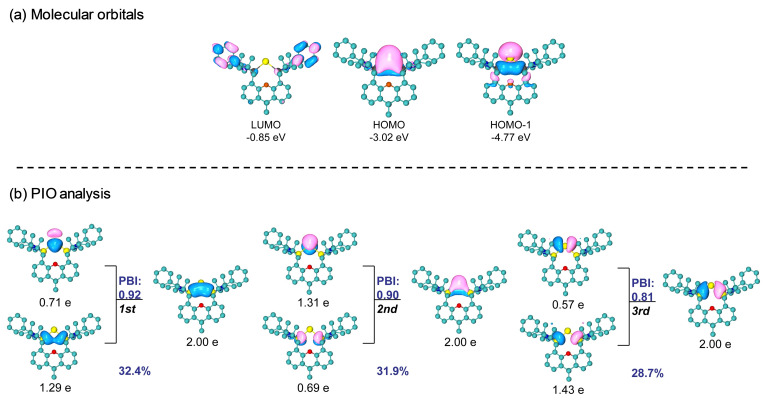
a) Molecular orbitals. b) PIO analysis on the bonding modes of Sn−Si in compound **5**. The compound **5** is decomposed to two moieties, the Sn atom and the rest. Hydrogen atoms in 3D structures are omitted for clarity. The total PBI value of two fragments is 2.83. The isosurface 0.050 au is plotted.

**Scheme 5:**
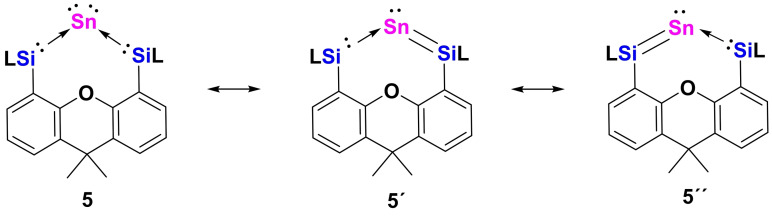
Proposed resonance structures of stannylone **5** [L=PhC(N*t*Bu)_2_].

In summary, while the common reductive approach to access a monoatomic zero‐valent Group 14 compounds failed in the case of the the Sn^II^ halide precursors {[Si^II^(Xant)Si^II^]Sn^II^Cl}Cl **2** and {[Si^II^(Xant)Si^II^]Sn^II^Br_2_} **3**, we could accomplish an iron‐mediated reduction of Sn^II^ in **2** and **3** to Sn^0^ in the stannylone‐iron complex {[Si^II^(Xant)Si^II^]Sn^0^[Fe(CO)_4_]_2_} **4** using K_2_Fe(CO)_4_. Reductive elimination of the [Fe(CO)_4_] groups of **4**, in turn, with KC_8_ enabled the synthesis and isolatation of the first bis(silylene)‐stabilized stannylone **5**. Its electronic structure and presence of a genuine two‐coordinate Sn^0^ center have been elucidated by DFT calculations. The HOMO and HOMO‐1 of **5** correspond to a π‐type lone pair, with delocalization into both adjacent Si^II^ vacant orbitals, and a σ‐type lone pair at the Sn^0^ center, respectively, indicating a genuine stannylone character. Further investigations on the reactivity of **5** are currently in progress.

## Conflict of interest

The authors declare no conflict of interest.

## Supporting information

As a service to our authors and readers, this journal provides supporting information supplied by the authors. Such materials are peer reviewed and may be re‐organized for online delivery, but are not copy‐edited or typeset. Technical support issues arising from supporting information (other than missing files) should be addressed to the authors.

Supporting Information
