# Supplementary material for: A Genuine Stannylone with a Monoatomic Two‐Coordinate Tin(0) Atom Supported by a Bis(silylene) Ligand
Source: Angew Chem Int Ed Engl. 2021 Dec 1;61(3):e202114073. doi: 10.1002/anie.202114073 (PMC9300062; doi:10.1002/anie.202114073)
Supplement: Supplementary file 1 — Supporting Information [file ANIE-61-0-s001.pdf]

Angewandte Chemie
Eine Zeitschrift der Gesellschaft Deutscher Chemiker

Supporting Information

A Genuine Stannylone with a Monoatomic Two-Coordinate Tin(0) Atom Supported by a Bis(silylene) Ligand

*J. Xu, C. Dai, S. Yao, J. Zhu, M. Driess**

Table of Content

A. Experimental Procedures 1
A1. General Considerations 1
A2. Single-Crystal X-ray Structure Determination........ 1
A3. Synthesis and Characterization 2
A4. NMR Spectra 3
A5. IR Spectra........ 12
A6. UV/Vis Spectra 14
A7. X-ray Crystallographic Data 15
B. DFT Calculations 23
C. TD-DFT Calculations 37
References........ 38

A. Experimental Procedures

A1. General Considerations

All experiments were carried out under dry oxygen-free nitrogen using standard Schlenk techniques or MBraun glove box fitted with a gas purification and recirculation unit. Solvents were dried by standard methods and freshly distilled prior to use. Potassium graphite (KC_8) was prepared by reacting potassium with previously dried graphite in a 1:8 molar ratio at 160 °C for 2 h under dried nitrogen. Bis(NHSi)xanthene Si^{II}(Xant)Si^{II} **1**[1] [Si^{II}(Xant)Si^{II} = PhC(N*t*Bu)$_2$Si(Xant)Si(N*t*Bu)$_2$CPh] and $K_2Fe(CO)_4$[2] were synthesized according to reported procedures. The solution NMR spectra were recorded on Bruker Spectrometers AV 200, 400 or 500 with residual solvent signals as internal reference (^{1}H NMR: D_6-Benzene, 7.16 ppm; ^{13}C{^{1}H} NMR: D6-Benzene, 128.06 ppm) or external standards (^{29}Si{^{1}H} NMR: $SiMe_4$, 0.0 ppm; ^{119}Sn{^{1}H} NMR: $SnMe_4$, 0.0 ppm). The following abbreviations were used to describe peak patterns when appropriate: *br* = broad, *s* = singlet, *d* = doublet, *t* = triplet, *dd* = doublet of doublets, *m* = multiplet. Elemental analyses were performed by the analytical labor service in the Institute of Chemistry, Technical University of Berlin, Germany. High-resolution ESI-MS were measured on a Thermo Scientific LTQ orbitrap XL. UV/Vis spectra were recorded on an Analytik Jena Specord S600 diode array spectrometer. IR spectra were measured with a Nicolet iS5 FT-IR-Spectrometer from the company Thermo.

A2. Single-Crystal X-ray Structure Determination

Crystals were each mounted on a glass capillary in perfluorinated oil and measured in a cold N_2 flow. The data of all compounds were collected on an Oxford Diffraction SuperNova, Single source at offset, Atlas at 150 K (Cu-Kα radiation, λ = 1.54184 Å). The structures were solved by direct methods and refined on F^2 with the SHELX-97 software package.[3] For the crystal of compound **4**, the strongly disordered C_6H_6 molecules are treated using Solvent Masking in Olex2. In the molecular structure of compound **5** which contains two independent molecules ***a*** and ***b***, one of the *tert*-butyl groups in molecule ***a*** is disordered over two orientations with an approximate occupancy ratio of 0.75:0.25; two of the *tert*-butyl groups in molecule ***b*** are disordered over two orientations with an approximate occupancy ratio of 0.75:0.25 and 0.72:0.28, respectively. **CCDC:** 2110196 (**2**), 2110197 (**3**), 2110198 (**4**) and 2110199(**5**) contain the supplementary crystallographic data for this paper. These data can be obtained free of charge from The Cambridge Crystallographic Data Centre via www.ccdc.cam.ac.uk/structures/

A3. Synthesis and Characterization

Synthesis of Compound 2.
To a mixture of bis(NHSi)xanthene **1** (728 mg, 1 mmol) and $SnCl_2$(dioxane) (277 mg, 1 mmol) in a 50 mL Schlenk flask was added 25 mL Et_2O at room temperature under stirring. A bright yellow precipitate formed slowly. After stirring overnight, the bright yellow precipitate was separated by filtration and dried under vacuum affording compound **2** as a yellow solid (780 mg, 85% isolated yields). Yellow crystals suitable for X-ray analysis were obtained from a concentrated toluene solution at -30 °C.

2: M.p. 271 °C (decomp.). ^{1}H NMR (200 MHz, D_6-Benzene, 298 K): δ = 7.82 (dd, *J* = 6.9, 1.9 Hz, 2 H, Ar(Xant)-*H*), 7.44 – 7.36 (m, 4 H, Ar(Ph)-*H*), 7.40 – 7.35 (m, 2 H, Ar(Xant)-*H*), 7.10 – 7.05 (m, 2 H, Ar(Xant)-*H*), 6.85 – 6.93 (m, 6 H, Ar(Ph)-*H*), 1.48 (s, 6 H, C(C*H*$_3$)$_2$), 1.36 ppm (s, 36 H, NC(C*H*$_3$)$_3$). ^{13}C{^{1}H} NMR (50 MHz, D_6-Benzene, 298 K): δ = 173.01(s, N*C*N), 158.25, 132.31, 131.81, 131.05, 130.59, 129.95, 129.16, 128.34, 128.06, 127.87, 124.05, 123.66(s, Ar-*C*) [A signal for Ar-*C* is covered by those of C_6D_6], 55.40 (s, N*C*(CH$_3$)$_3$), 31.72 (s, C(*C*H$_3$)$_2$), 31.57 (s, NC(*C*H$_3$)$_3$), 29.92 ppm (s, *C*(CH$_3$)$_2$). ^{119}Sn{^{1}H} NMR (149 MHz, D_6-Benzene, 298 K): δ = -348.13 ppm. ^{29}Si{^{1}H} NMR (79 MHz, D_6-Benzene, 298 K): δ = 29.36 ppm ($^1J_{Si, Sn}$ = 1334 Hz). HRMS(ESI): (m/z) calcd for $(C_{45}H_{58}ClN_4OSi_2Sn)^+$: 881.2854 [M-Cl]$^+$; found: 881.2825. Elemental analysis calcd for $C_{45}H_{58}Cl_2N_4OSi_2Sn$: C, 58.96; H, 6.38; N, 6.11; found: C, 56.85; H, 6.34; N, 5.65 [Consistently low C analysis may be due to the formation of silicon carbide]. IR (cm^{-1}): 2969(w), 1472(w), 1445(w), 1395(s), 1368(m), 1274(w), 1239(m), 1200(m), 1120(w), 1089(w), 1020(w), 801(w), 780(m), 763(m), 746(m), 726(w), 717(w), 709(m), 630(m).

Synthesis of Compound 3.
To a mixture of bis(NHSi)xanthene **1** (728 mg, 1 mmol) and $SnBr_2$(dioxane) (467 mg, 1 mmol) in a 50 mL Schlenk flask was added 25 mL Et_2O at room temperature under stirring. A bright yellow precipitate formed slowly. After stirring overnight, the bright yellow precipitate was separated by filtration and dried under vacuum affording compound **3** as yellow solid (734mg, 72% isolated yields). Yellow crystals suitable for X-ray analysis were obtained from a concentrated Et_2O solution at -30 °C.

3: M.p. 284 °C (decomp.). ^{1}H NMR (200 MHz, D_6-Benzene, 298 K) δ = 7.79 (dd, *J* = 7.1, 1.7 Hz, 2H, Ar(Xant)-*H*),), 7.68 (d, *J* = 8.3 Hz, 2H, Ar(Xant)-*H*), 7.41 (m, 2H, Ar(Ph)-*H*), 7.33 – 7.24 (m, 2H, Ar(Xant)-*H*), 7.03 – 6.81 (m, 8H, Ar(Ph)-*H*), 1.48 (s, 6H, C(C*H*$_3$)$_2$), 1.37 ppm(s, 36H, NC(C*H*$_3$)$_3$). ^{13}C{^{1}H} NMR (50 MHz, D_6-Benzene, 298 K) δ = 173.34 (s, N*C*N), 158.09, 131.83, 131.75, 130.93, 130.58, 130.51, 128.81, 128.35, 127.86,123.76, 122.95 (s, Ar-*C*) [A signal for Ar-*C* is covered by those of C_6D_6], 55.46 (s, N*C*(CH$_3$)$_3$), 35.65 (s, C(*C*H$_3$)$_2$), 31.95 (s, NC(*C*H$_3$)$_3$), 30.36 ppm (s, *C*(*C*H$_3$)$_2$).^{119}Sn{^{1}H} NMR (75 MHz, D_6-Benzene, 298 K) δ = -391.95ppm. ^{29}Si{^{1}H} NMR (99 MHz, D_6-Benzene, 298 K) δ = 30.27 ppm ($^1J_{Si, Sn}$ = 1350 HZ). HRMS(ESI): (m/z) calcd for $(C_{45}H_{58}BrN_4OSi_2Sn)^+$: 925.2349 [M-Br]$^+$; found: 925.2316. Elemental analysis calcd for $C_{45}H_{58}Br_2N_4OSi_2Sn$: C, 53.74; H, 5.81; N, 5.57; found: C, 48.42; H, 5.37; N, 4.70 [Consistently low C analysis may be due to the formation of silicon carbide]. IR (cm^{-1}): 2969(w), 1471(w), 1395(s), 1362(m), 1272(w), 1235(m), 1199(m), 1118(w), 1105(w), 1089(w), 1079(w), 1021(w), 860(w), 801(w), 780(m), 763(m), 745(m), 726(w), 715(w), 709(m), 630(m), 614(w), 574(w), 563(w).

Synthesis of Compound 4.

Reduction of 2: To a mixture of compound **2** (917 mg, 1 mmol) and $K_2Fe(CO)_4$ (295.2mg, 1.2 mmol) in a 100 mL Schlenk flask was added 60 mL THF at room temperature under stirring. The color of the mixture changed to red immediately. After stirring overnight, the red mixture was filtered and the residue was washed with THF (10 mL x 2). Volatiles were removed under vacuum and the residue was washed with Et_2O (10 mL) to afford compound **4** as a red powder after dried under vacuum (496 mg, 42% isolated yield). Red crystals suitable for X-ray analysis were obtained from a concentrated benzene solution at room temperature.

Reduction of 3: To a mixture of compound **3** (100.7 mg, 0.1 mmol) and $K_2Fe(CO)_4$ (29.52mg, 0.12 mmol) in a 25 mL Schlenk flask was added 10 mL THF at room temperature under stirring. The color of the mixture changed to red immediately. After stirring overnight, the red mixture was filtered and the residue was washed with THF (2 mL x 2). Volatiles were removed under vacuum and the residue was washed with Et_2O (3 mL) to afford compound **4** as a red powder after dried under vacuum (47 mg, 40% isolated yield).

4: M.p. 214 °C(decomp.). ^{1}H NMR (200 MHz, D_6-Benzene, 298 K) δ = 7.93 – 7.86 (m, 2H, Ar(Xant)-*H*), 7.67 (dd, *J* = 7.1, 1.7 Hz, 2H, Ar(Xant)-*H*), 7.30 (d, *J* = 1.7 Hz, 2H, Ar(Xant)-*H*), 7.21 (m, 4H, Ar(Ph)-*H*), 7.00 – 6.92 (m, 6H, Ar(Ph)-*H*), 1.43 (s, 6H, $C(CH_3)_2$), 1.21 ppm (s, 36H, $NC(CH_3)_3$). ^{13}C{^{1}H} NMR (50 MHz, D_6-Benzene, 298 K) δ = 221.72 (s, *C*=O), 172.70 (s, N*C*N), 158.55, 132.30, 131.74, 130.54, 129.74, 129.17, 128.93, 128.35, 127.85, 127.35, 124.94, 123.72 (s, Ar-*C*), 55.35 (s, N*C*($CH_3)_3$), 35.46 (s, $C(CH_3)_2$, 31.48 (s, $NC(CH_3)_3$), 30.24 ppm (s, $C(CH_3)_2$).^{119}Sn{^{1}H} NMR (149 MHz, D_6-Benzene, 298 K) δ = 7.29 ppm. ^{29}Si{^{1}H} NMR (79 MHz, D_6-Benzene, 298 K) δ = 28.08 ppm [The solubility in C_6D_6 is not good enough, no $^{119/117}$Sn satellites were observed]. ^{29}Si{^{1}H} NMR (79 MHz, D_8-THF, 298 K) δ = 27.00 ppm ($^1J_{Si,\ Sn}$ = 1312 HZ). HRMS(ESI): (m/z) calcd for $(C_{49}H_{59}N_4O_5Si_2FeSn)^+$: 1015.2390 [M-Fe(CO)$_4$+H]$^+$; found: 1015.2412. Elemental analysis calcd for $C_{53}H_{58}N_4O_9Si_2Fe_2Sn$: C, 53.87; H, 4.95; N, 4.74 found: C, 52.46; H, 5.82; N, 4.98. UV-Vis (toluene), λ_{max} (ε_{max}/(M^{-1} cm^{-1}): 493 nm (6229). IR (cm^{-1}): 2970(w), 1962(s), 1881(m), 1834(s), 1427(m), 1459(w), 1394(s), 1366(m), 1276(w), 1249(w), 1238(w), 1224(w), 1201(m), 1120(w), 1089(w),1023(w), 785(w), 764(m), 746(w), 724(w), 706(m), 619(s).

Synthesis of Compound 5. To a mixture of compound **4** (591.5 mg, 0.5 mmol) and KC_8 (276.75 mg, 2.05 mmol) in a 100 mL Schlenk flask was added 50 mL THF at room temperature under stirring. The color of the mixture from red changed to dark blue slowly. After stirring 6 h, the dark blue mixture was filtered and the black residue of graphite was washed with THF (10 mL x 3). The volatiles were removed under vacuum from the combined filtrate to give blue residue. Then hexane (80 mL) was introduced to the residue, the resulting solution was separated from the precipitates by filtration. The volatiles were removed under vacuum to afford almost pure compound **5** as a dark blue powder (474.32mg, 56% isolated yields). Dark blue crystal suitable for X-ray analysis was obtained from a saturated isopropyl ether solution at -20°C more than 5 days.

5: M.p. 198 °C (decomp.). ^{1}H NMR (200 MHz, D_6-Benzene, 298 K) δ = 7.73 (dd, *J* = 6.1, 2.7 Hz, 2H, Ar(Xant)-*H*),), 7.27 – 7.22 (m, 4H, Ar(Xant)-*H*), 7.12 – 7.07 (m, 2H, Ar(Ph)-*H*), 7.00 – 6.79 (m, 8H, Ar(Ph)-*H*), 1.53 (s, 6H, $C(CH_3)_2$), 1.42 ppm (s, 36H, $NC(CH_3)_3$). ^{13}C{^{1}H} NMR (50 MHz, D_6-Benzene, 298 K) δ = 168.14 (s, N*C*N), 159.65, 134.09, 133.08, 129.71, 129.51, 128.64, 128.35, 128.22, 127.84, 126.46, 125.00, 123.03 (s, Ar-*C*), 55.22 (s, N*C*($CH_3)_3$), 36.37 (s, $C(CH_3)_2$), 32.00 (s, $NC(CH_3)_3$), 28.92 (s, $C(CH_3)_2$). ^{119}Sn{^{1}H} NMR (149 MHz, D_6-Benzene, 298 K) δ = -1147.24 ppm. ^{29}Si{^{1}H} NMR (79 MHz, D_6-Benzene, 298 K) δ = 50.60 ppm ($^1J_{Si,\ Sn}$ = 1244 HZ). HRMS(ESI): (m/z)calcd for $(C_{45}H_{59}N_4OSi_2Sn)^+$: 847.3244 [M+H]$^+$; found: 847.3244. Elemental analysis calcd for $C_{45}H_{58}N_4OSi_2Sn$: C, 63.90; H, 6.91; N, 6.62; found: C, 61.95; H, 6.28; N, 7.02 [Consistently low C analysis may be due to the formation of silicon carbide]. UV-Vis (toluene), λ_{max} (ε_{max}/(M^{-1} cm^{-1}): 674 nm (3088). IR (cm^{-1}): 2963(w), 1472(w), 1391(s), 1361(m), 1273(w), 1205(m), 1081(w), 1022(w), 784(w), 749(s), 722(m), 705(s), 613(s).

Reaction of 5 with $Fe_2(CO)_9$. To a mixture of compound **5** (42.4 mg, 0.05 mmol) and $Fe_2(CO)_9$ (18.2mg, 0.05 mmol) was added 20 mL THF at room temperature under stirring. The color of the mixture changed to red immediately. After stirring overnight, volatiles were removed under vacuum and the residue was washed with Et_2O (10 mL) to afford compound **4** (47.3 mg, 80% isolated yields).

A4. NMR Spectra

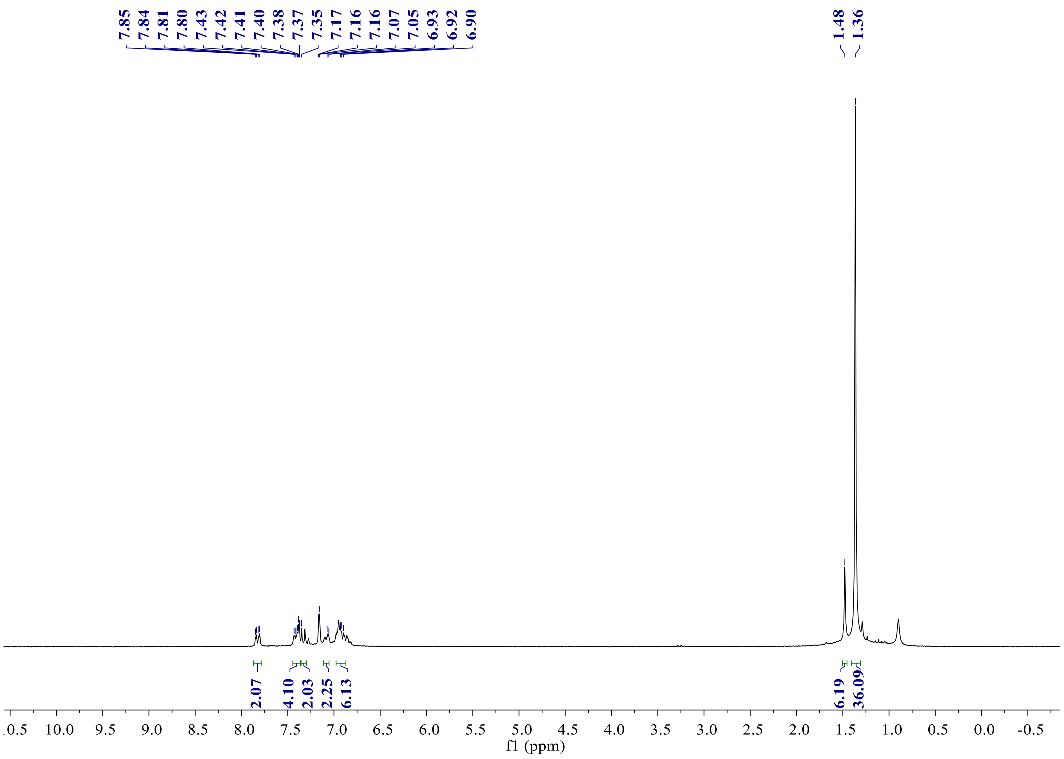

Figure S1. ^{1}H NMR spectrum of **2** in C_6D_6 under 1 bar N_2 at 298 K.

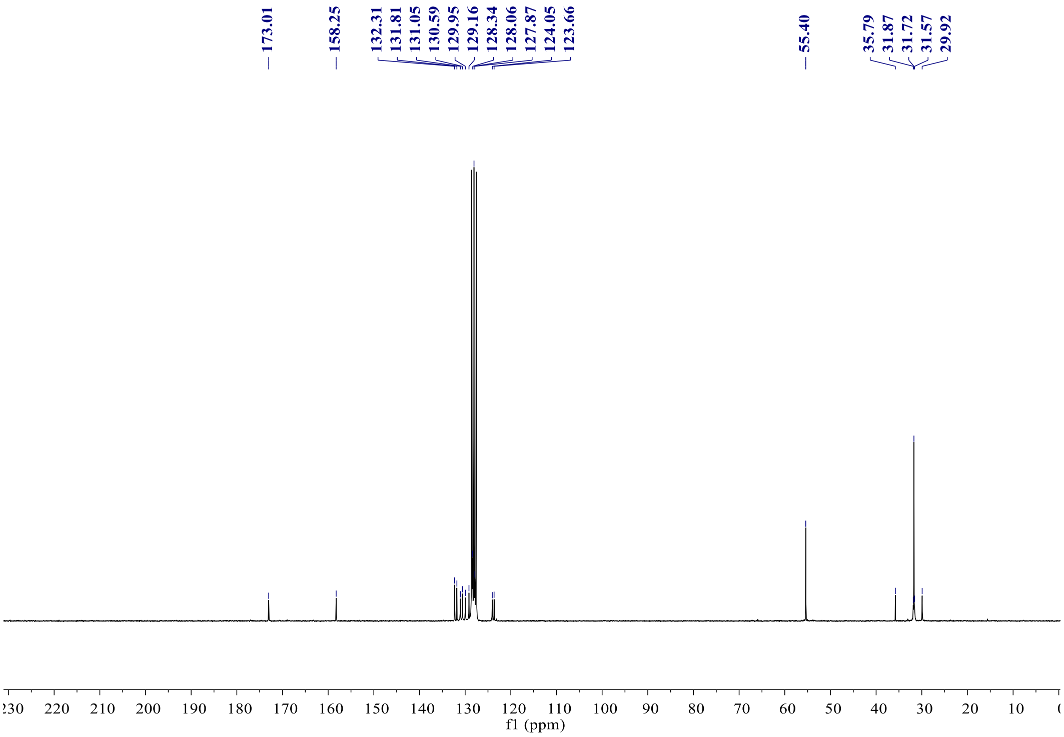

Figure S2. $^{13}C\{^{1}H\}$ NMR spectrum of **2** in C_6D_6 under 1 bar N_2 at 298 K.

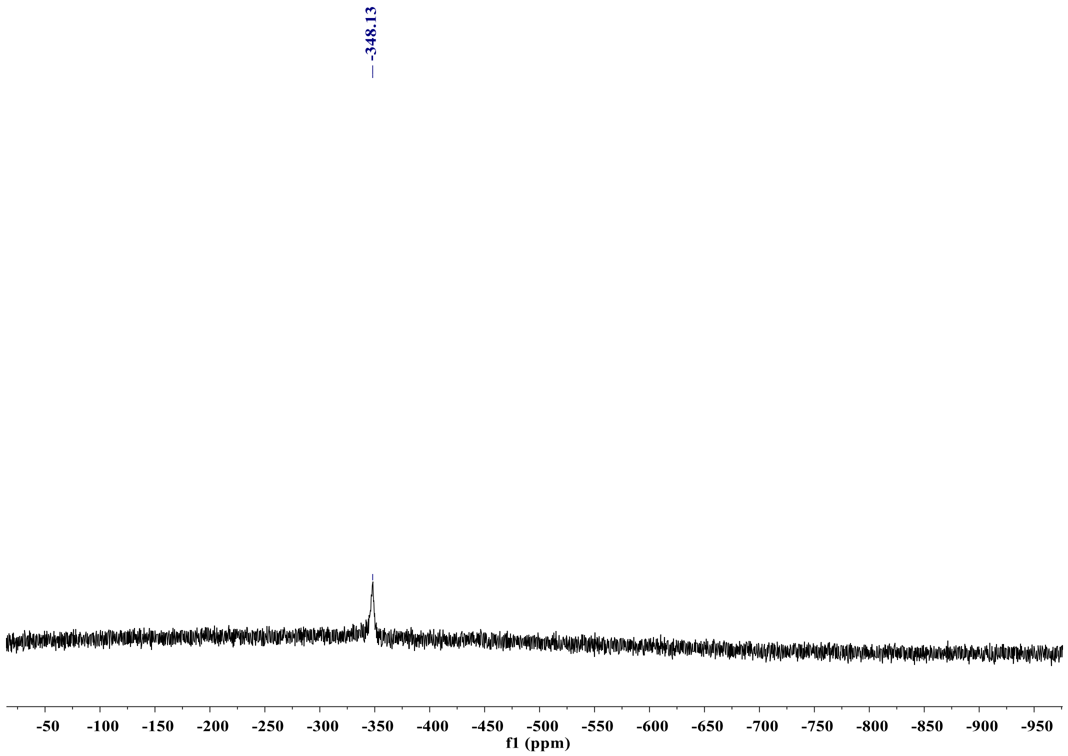

Figure S3. $^{119}Sn\{^{1}H\}$ NMR spectrum of **2** in C_6D_6 under 1 bar N_2 at 298 K.

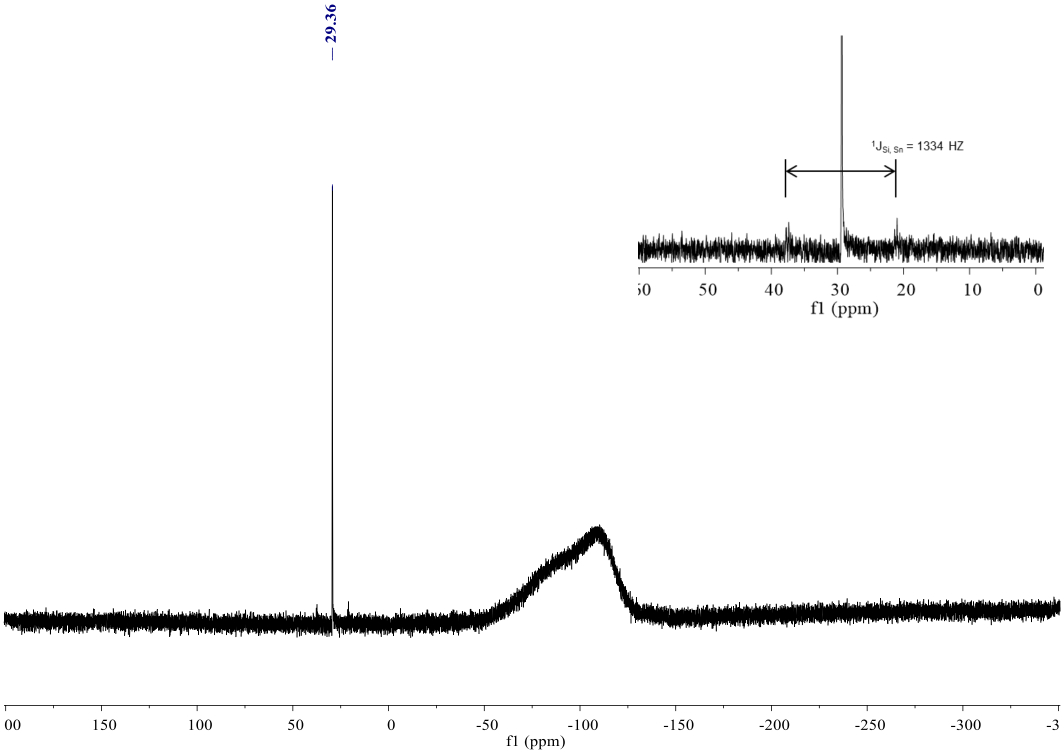

Figure S4. $^{29}Si\{^{1}H\}$ NMR spectrum of **2** in C_6D_6 under 1 bar N_2 at 298 K.

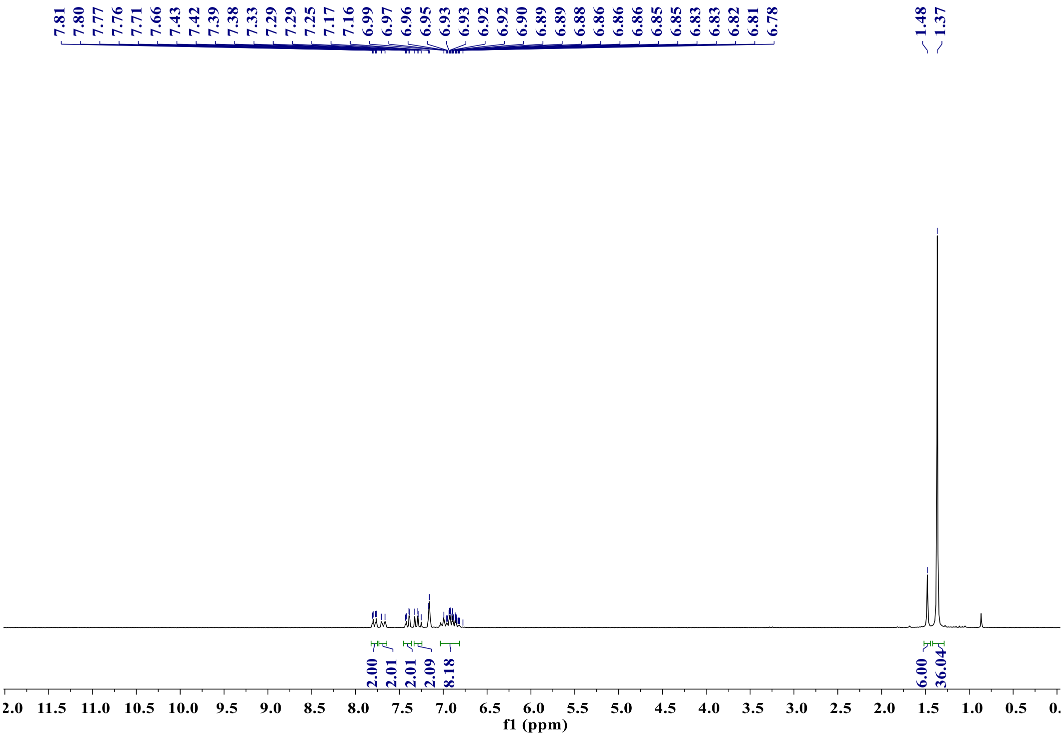

Figure S5. ^{1}H NMR spectrum of **3** in C_6D_6 under 1 bar N_2 at 298 K.

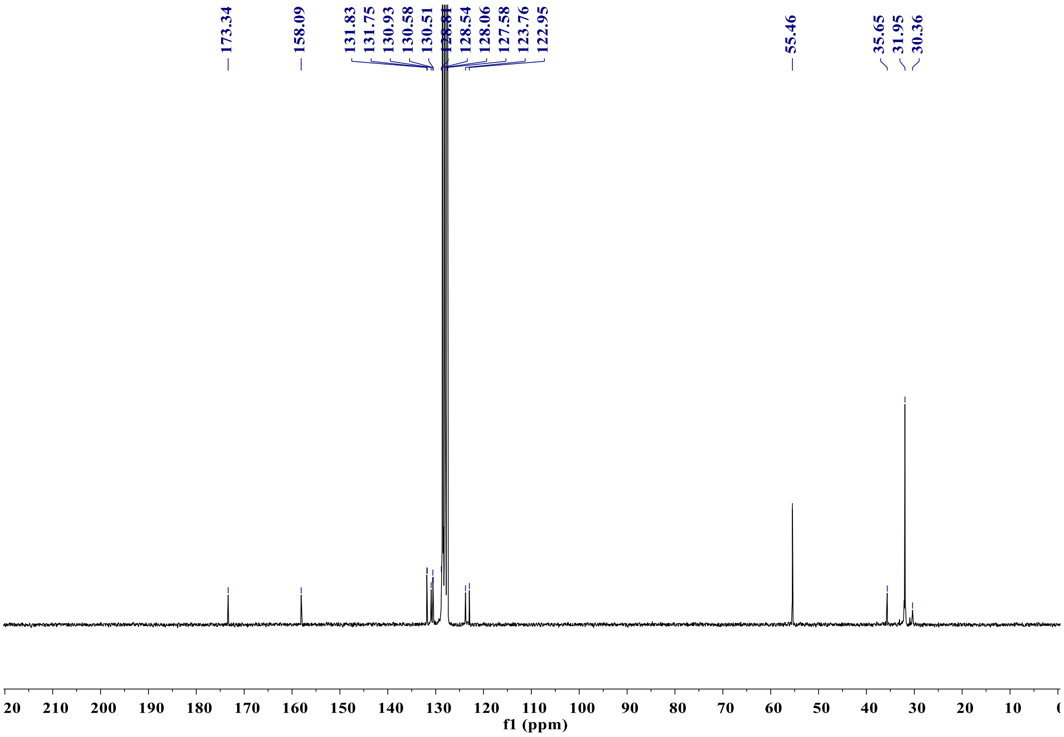

Figure S6. $^{13}C\{^{1}H\}$ NMR spectrum of **3** in C_6D_6 under 1 bar N_2 at 298 K.

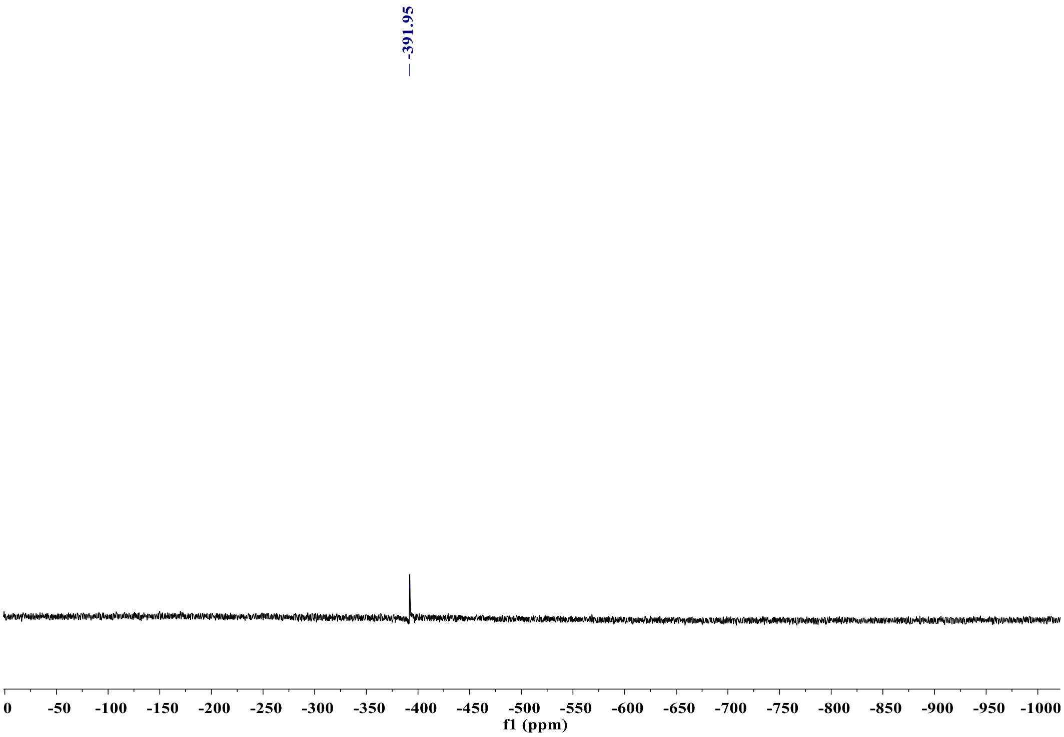

Figure S7. $^{119}Sn\{^1H\}$ NMR spectrum of **3** in C_6D_6 under 1 bar N_2 at 298 K.

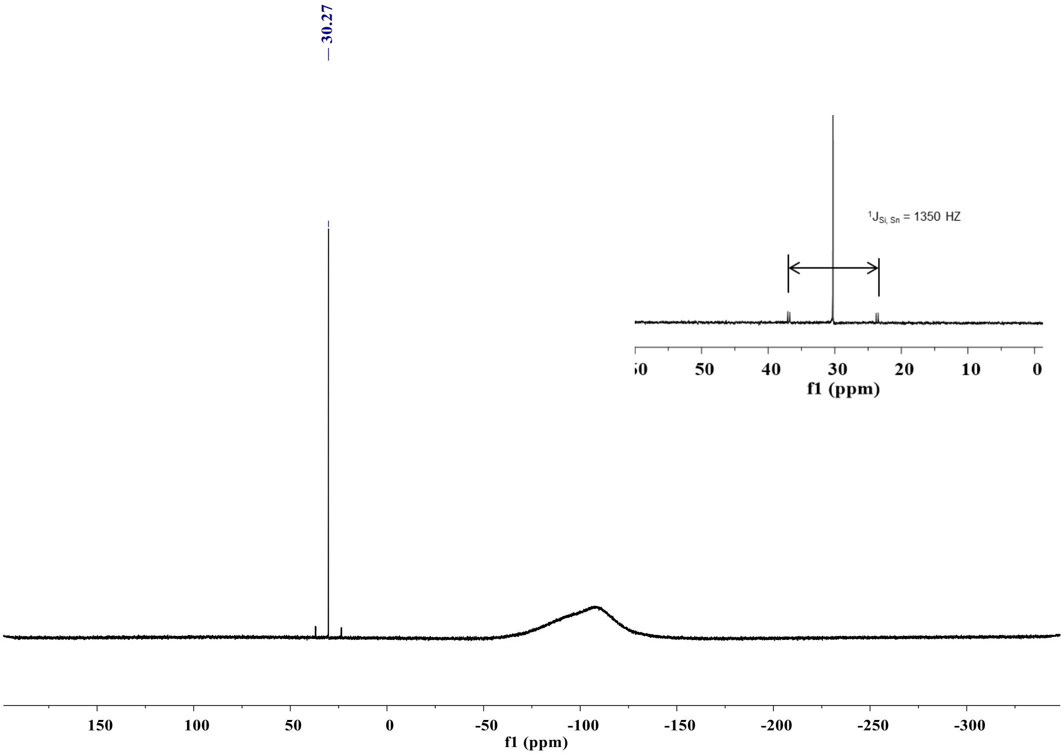

Figure S8. $^{29}Si\{^1H\}$ NMR spectrum of **3** in C_6D_6 under 1 bar N_2 at 298 K.

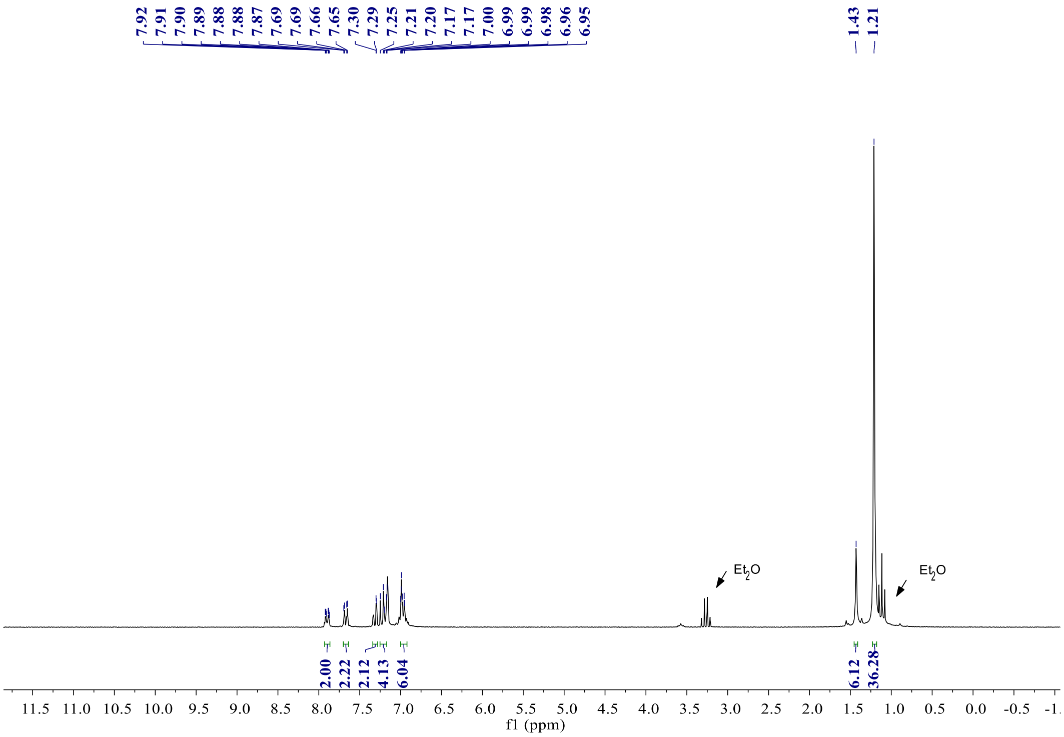

Figure S9. ^{1}H NMR spectrum of **4** in C_6D_6 under 1 bar N_2 at 298 K.

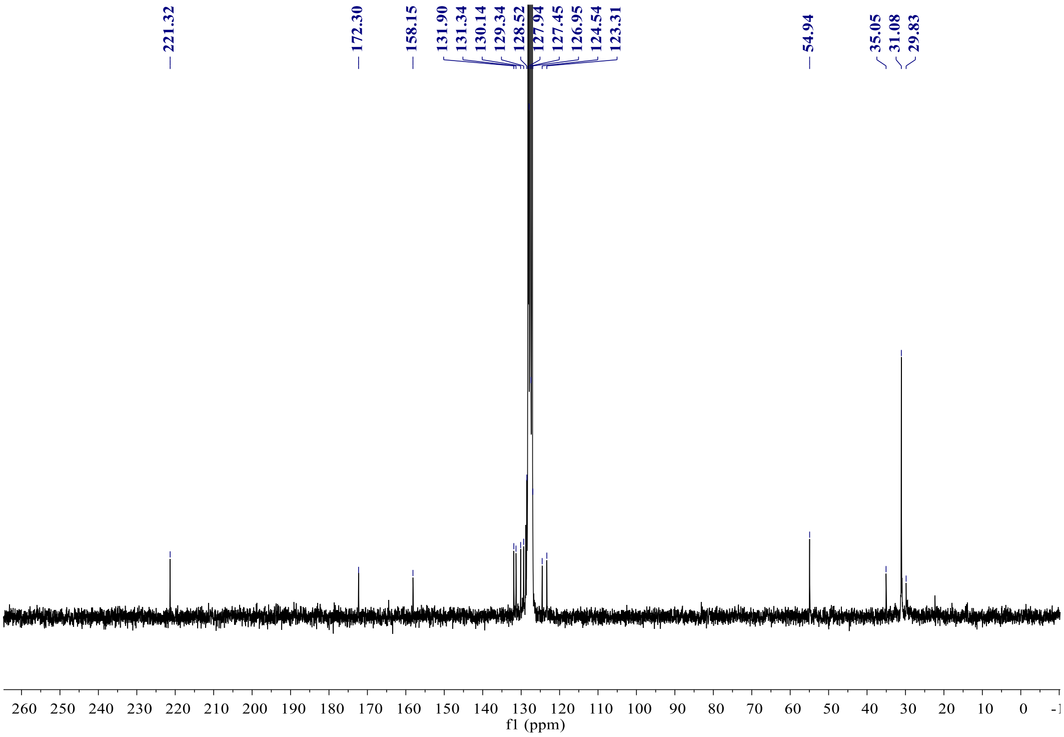

Figure S10. $^{13}C\{^{1}H\}$ NMR spectrum of **4** in C_6D_6 under 1 bar N_2 at 298 K.

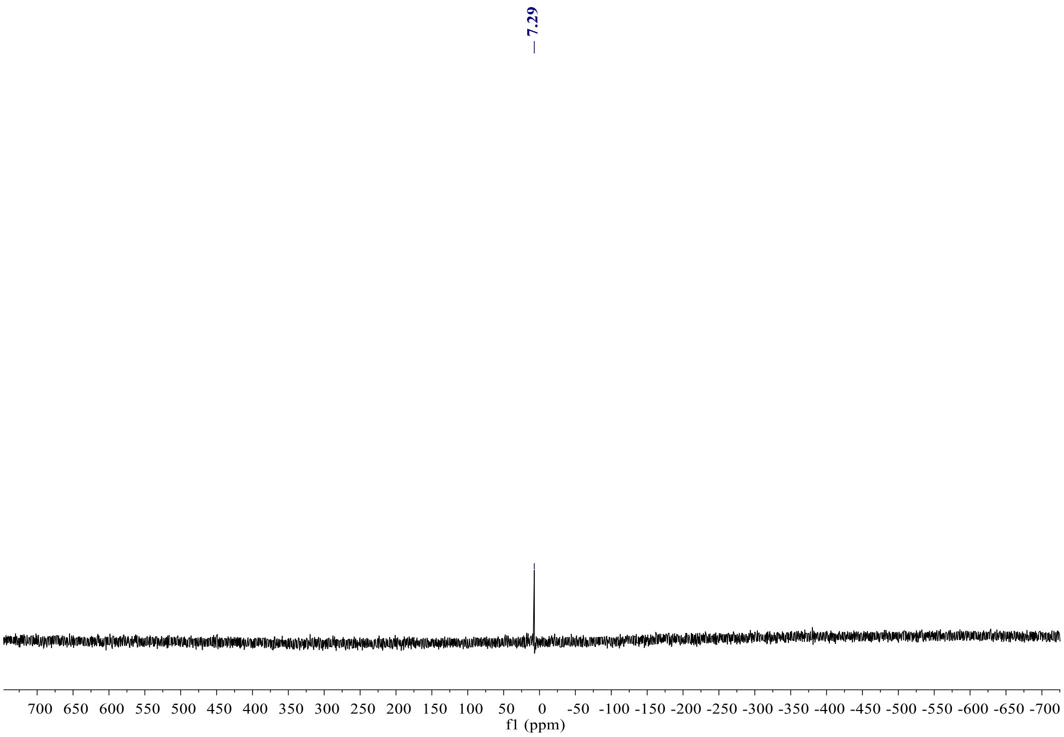

Figure S11. $^{119}Sn\{^{1}H\}$ NMR spectrum of **4** in C_6D_6 under 1 bar N_2 at 298 K.

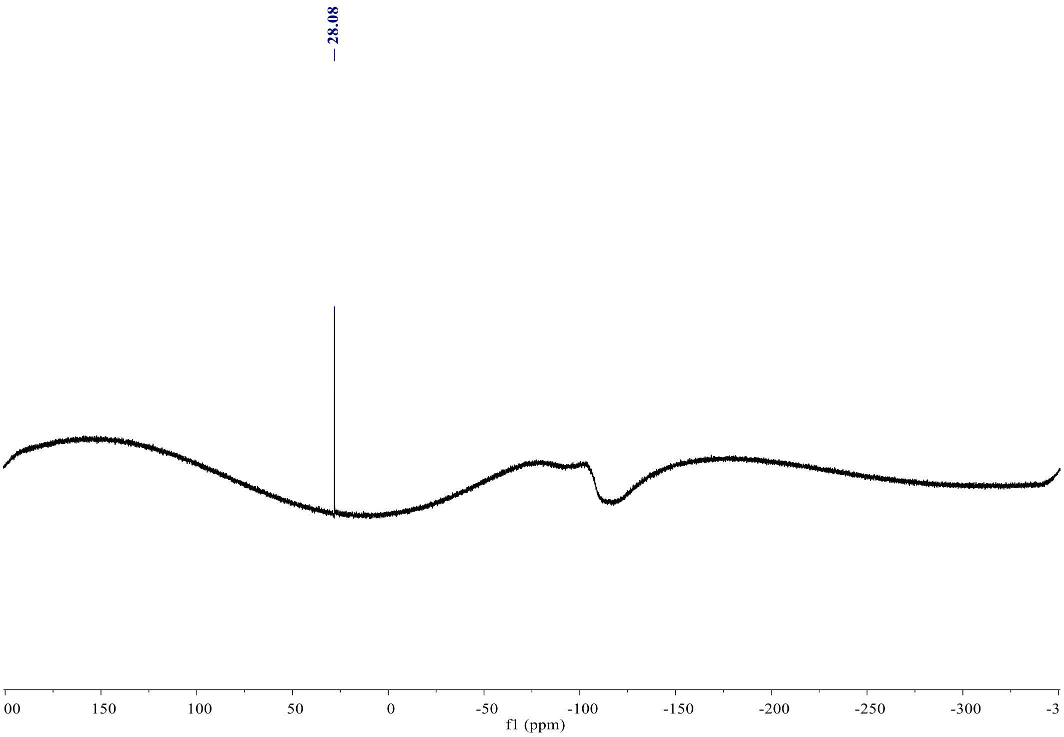

Figure S12(a). $^{29}Si\{^{1}H\}$ NMR spectrum of **4** in C_6D_6 under 1 bar N_2 at 298 K.

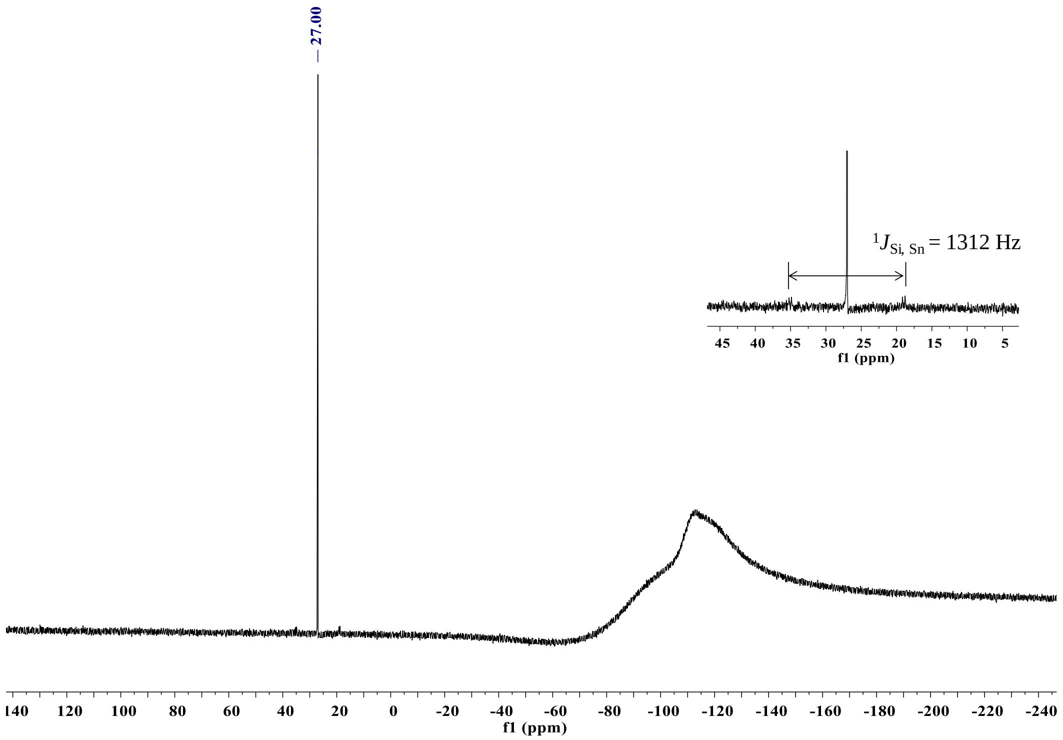

Figure S12(b). $^{29}Si\{^{1}H\}$ NMR spectrum of **4** in THF-*d8* under 1 bar N_2 at 298 K.

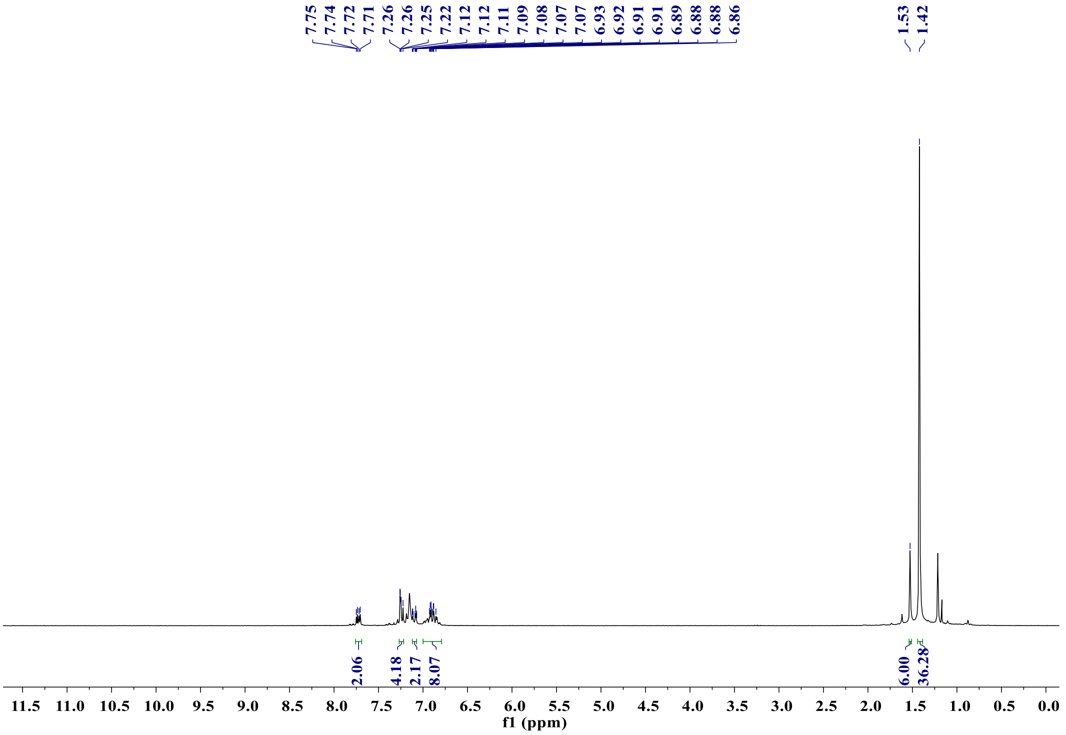

Figure S13. ^{1}H NMR spectrum of **5** in C_6D_6 under 1 bar N_2 at 298 K.

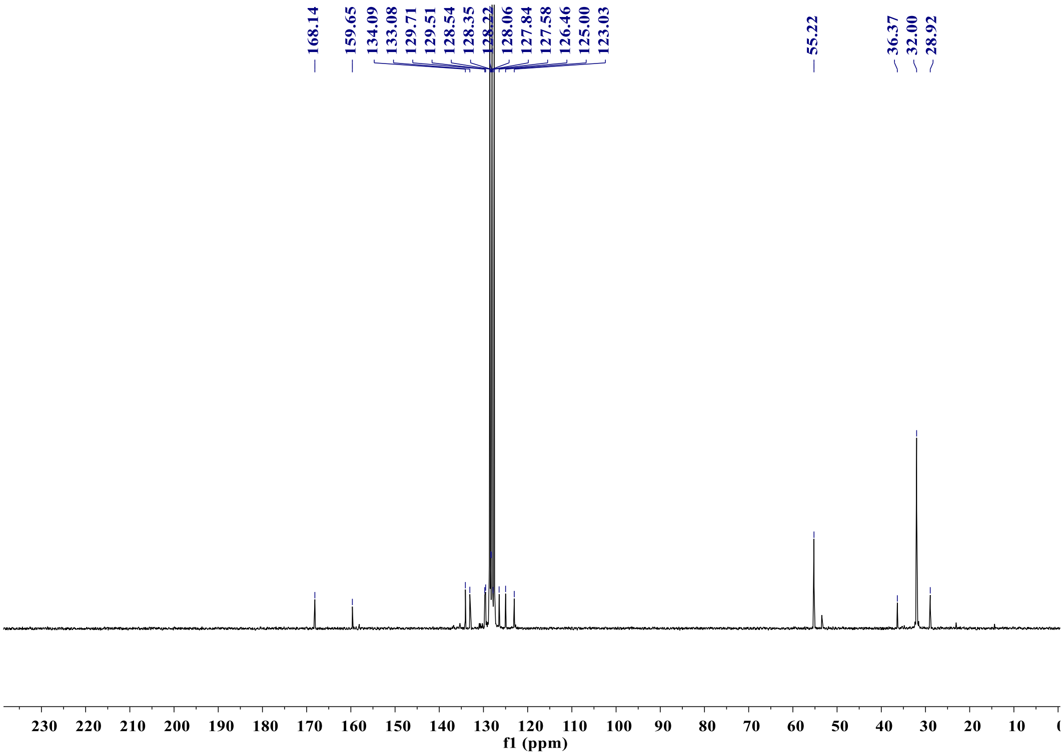

Figure S10. $^{13}C\{^{1}H\}$ NMR spectrum of **5** in C_6D_6 under 1 bar N_2 at 298 K.

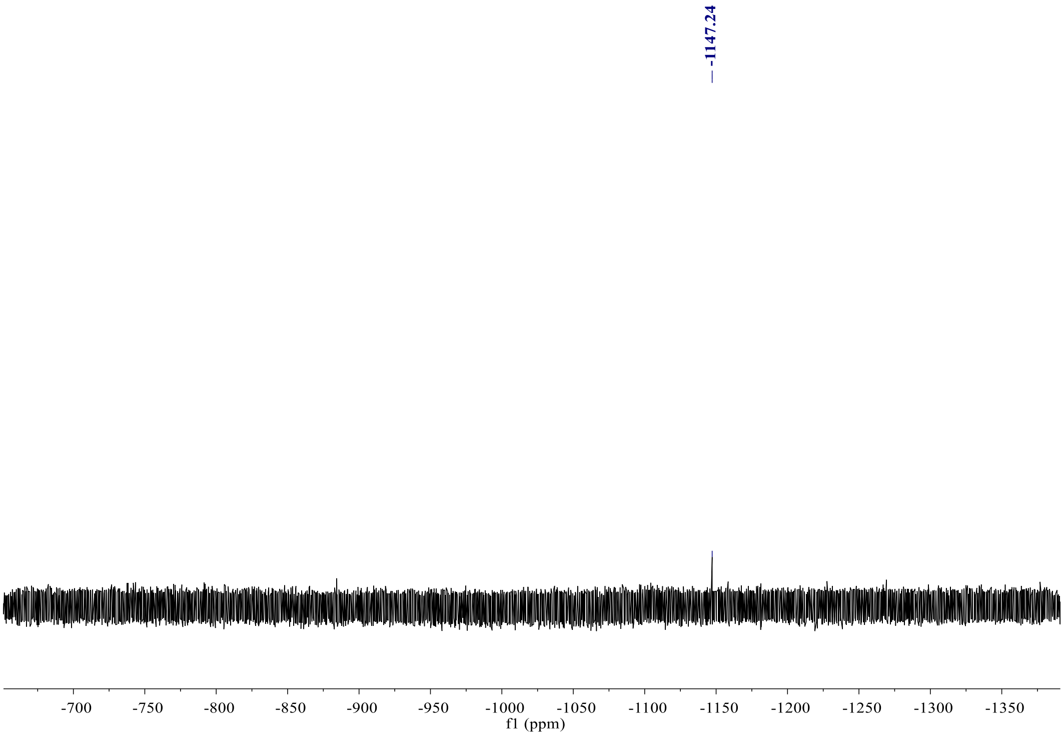

Figure S15. $^{119}Sn\{^{1}H\}$ NMR spectrum of **5** in C_6D_6 under 1 bar N_2 at 298 K.

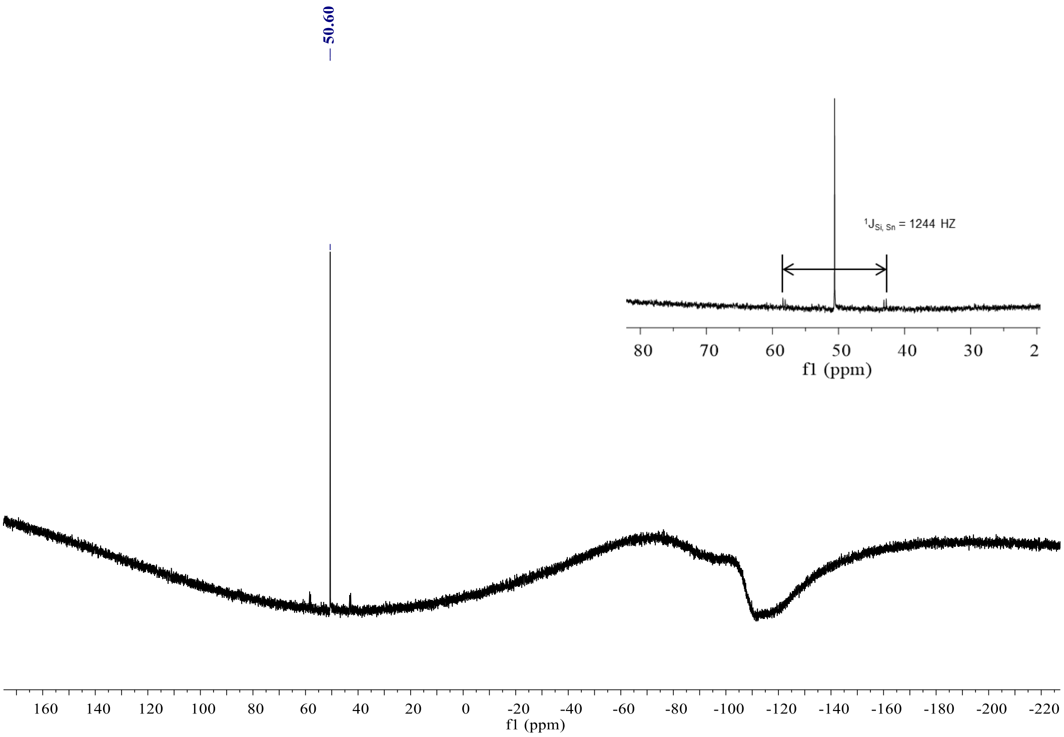

Figure S16. $^{29}Si\{^1H\}$ NMR spectrum of **5** in C_6D_6 under 1 bar N_2 at 298 K.

A5. IR Spectra

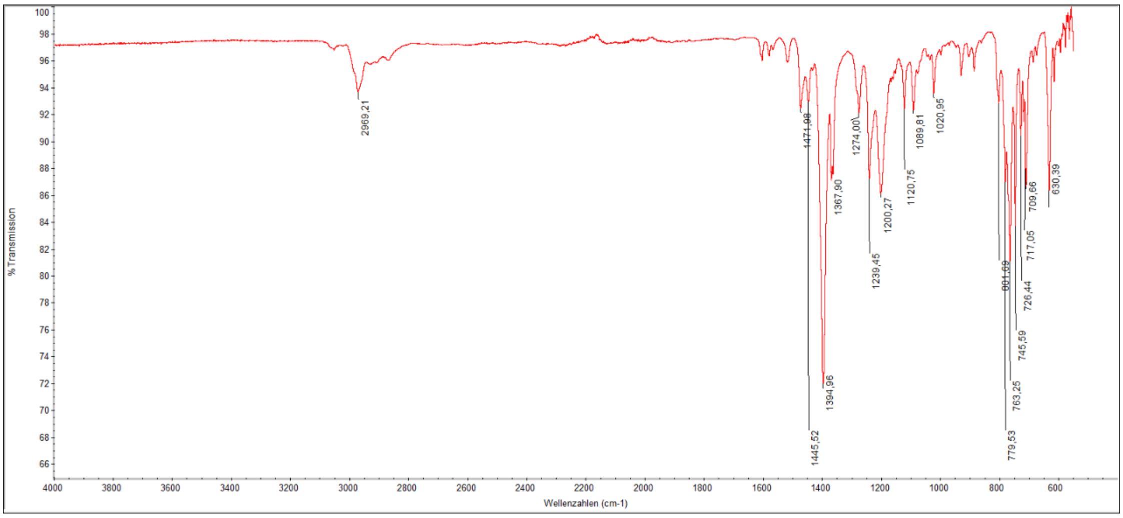

Figure S17. IR spectrum of **2**

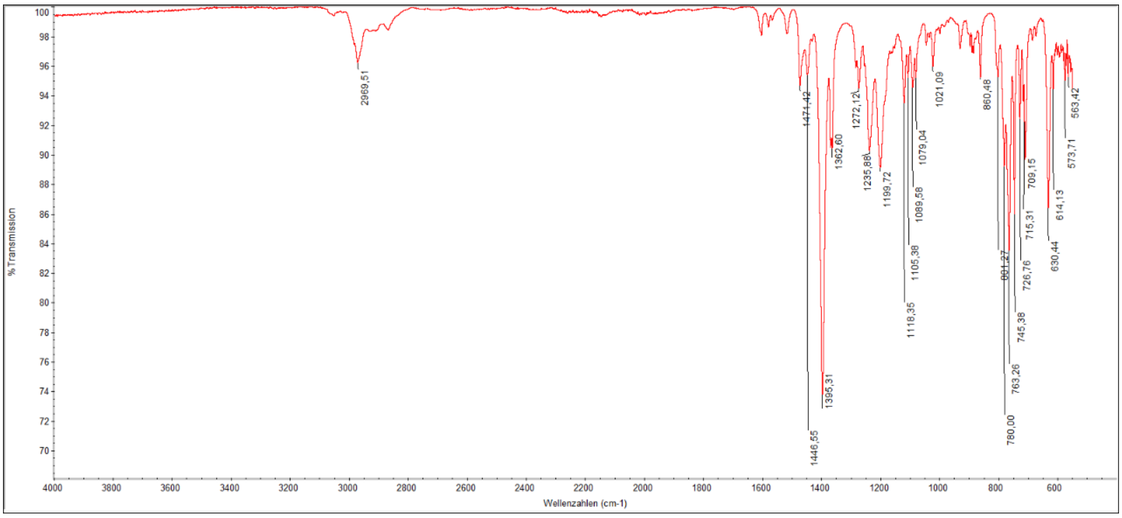

Figure S18. IR spectrum of **3**

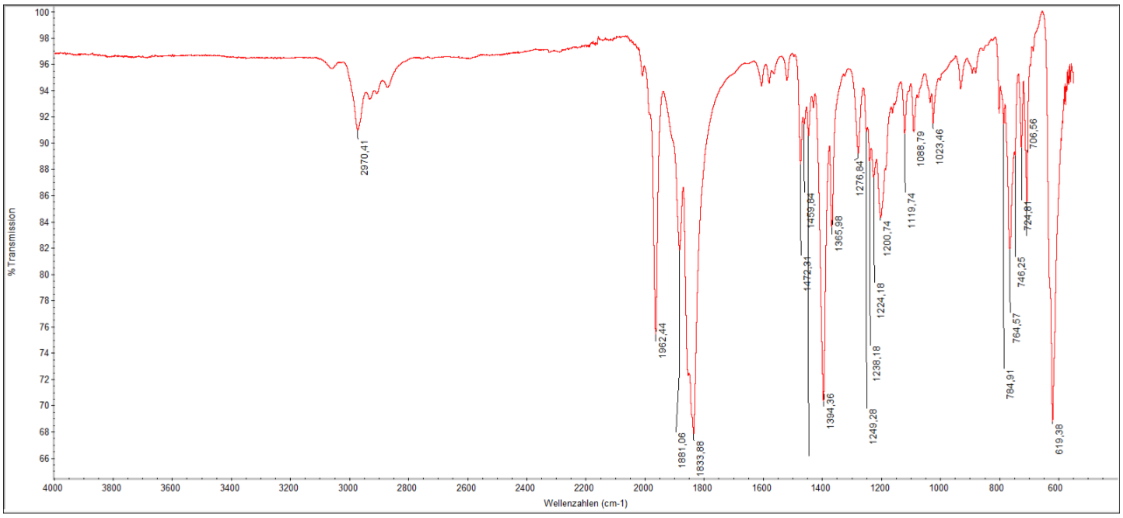

Figure S19. IR spectrum of **4**

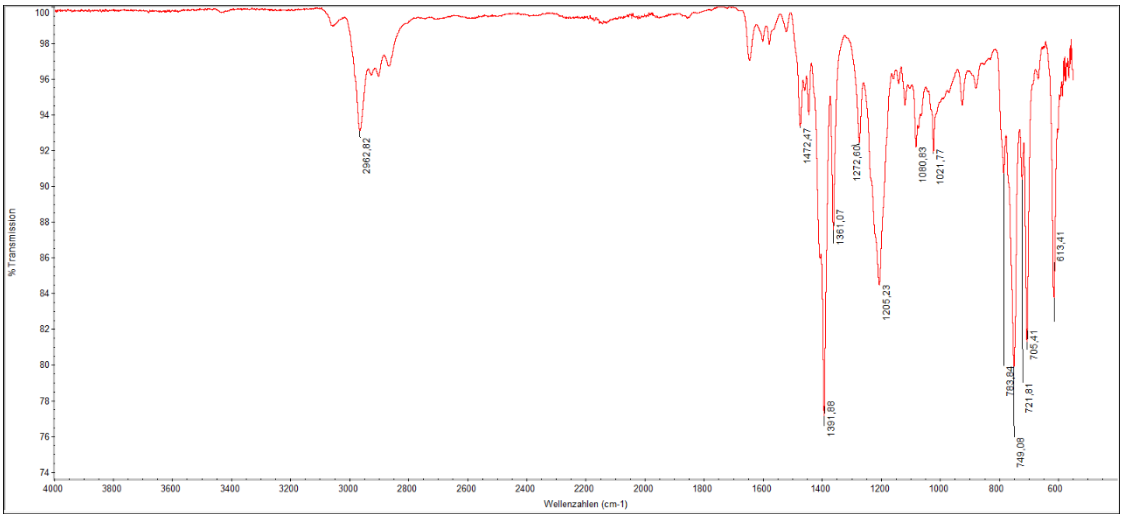

Figure S20. IR spectrum of **5**

A6. UV/Vis Spectra

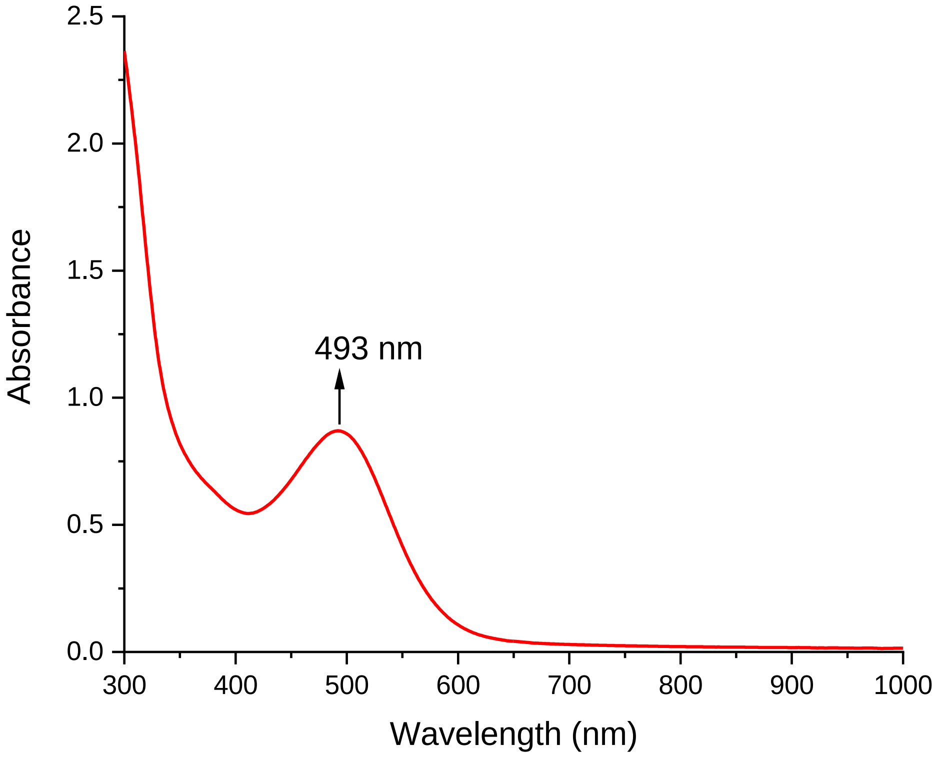

Figure S21. UV/Vis spectrum of compound **4** (RT, toluene, 1.4 x 10^{-4} M).

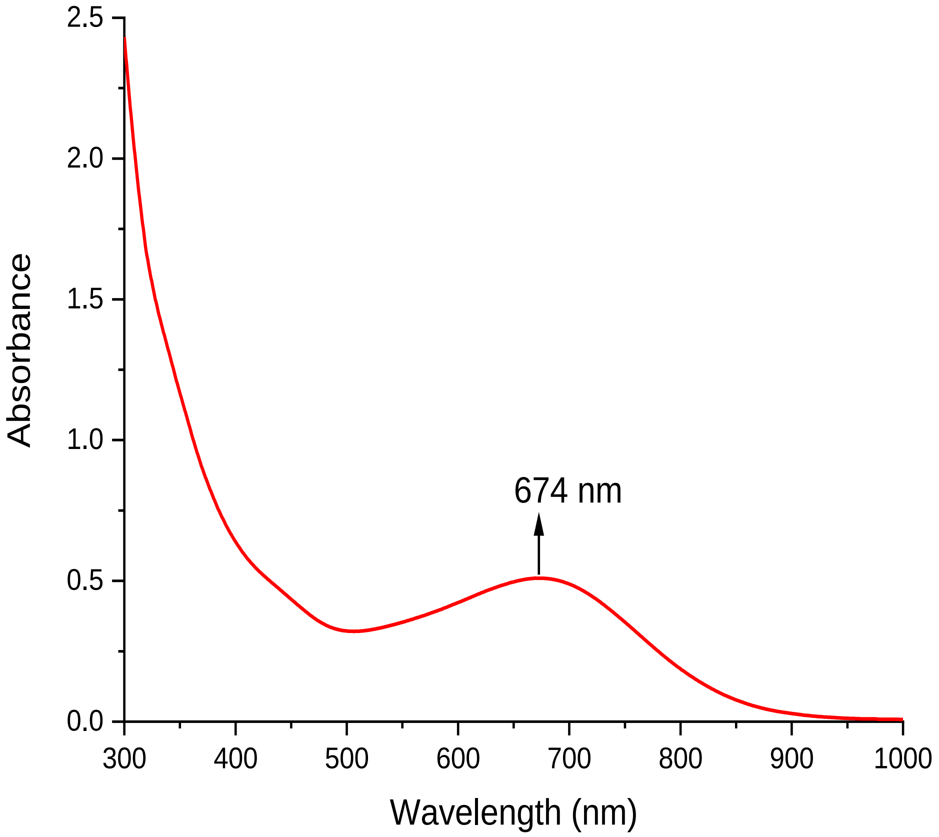

Figure S22. UV/Vis spectrum of compound **5** (RT, toluene, 1.65 x 10^{-4} M).

A7. X-ray Crystallographic Data

Table S1. Crystallographic data and structure refinement for compound **2**

Empirical formula	C52 H66 Cl2 N4 O Si2 Sn	
Formula weight	1008.85	
Temperature	150.00(10) K	
Wavelength	1.54184 Å	
Crystal system	Orthorhombic	
Space group	$Pca2_1$	
Unit cell dimensions	a = 18.6531(3) Å	a= 90°.
	b = 15.7277(2) Å	b= 90°.
	c = 17.5139(2) Å	g = 90°.
Volume	5138.06(12) Å^3	
Z	4	
Density (calculated)	1.304 Mg/m^3	
Absorption coefficient	5.662 mm^{-1}	
F(000)	2104	
Crystal size	0.02 x 0.02 x 0.01 mm^3	
Theta range for data collection	2.810 to 72.607°.	
Index ranges	-22<=h<=23, -19<=k<=17, -20<=l<=21	
Reflections collected	21196	
Independent reflections	8171 [R(int) = 0.0301]	
Completeness to theta = 67.684°	100.0 %	
Absorption correction	Semi-empirical from equivalents	
Max. and min. transmission	1.00000 and 0.53572	
Refinement method	Full-matrix least-squares on F^2	
Data / restraints / parameters	8171 / 1 / 574	
Goodness-of-fit on F^2	1.063	
Final R indices [I>2sigma(I)]	R1 = 0.0466, wR2 = 0.1180	
R indices (all data)	R1 = 0.0498, wR2 = 0.1222	
Absolute structure parameter	-0.026(7)	
Extinction coefficient	n/a	
Largest diff. peak and hole	1.059 and -1.553 e.Å^{-3}	

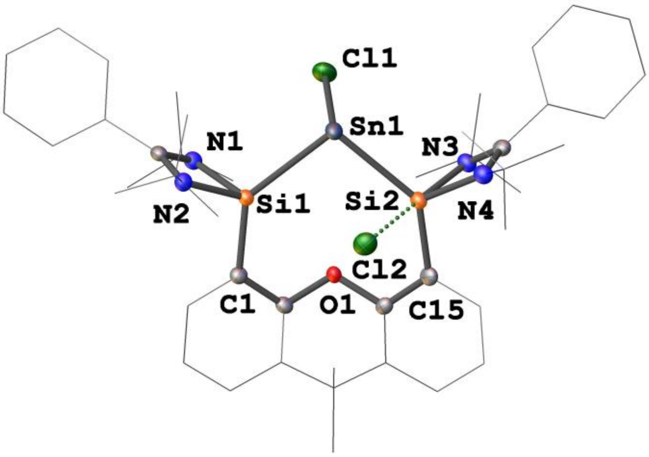

Figure **S23**. Molecular structure of compound **2**. Thermal ellipsoids are drawn at the 50% probability level. H atoms and solvent (toluene) molecules are omitted for clarity.

Table S2. Selected interatomic distances and angles of compound **2**.

Interatomic distances(Å)		Angles(°)	
Si(1)−Sn(1)	2.6747(15)	Si(1)−Sn(1)−Si(2)	99.62(5)
Si(2)−Sn(1)	2.6216(17)	Si(1)−Sn(1)−Cl(1)	91.85(5)
Sn(1)−Cl(1)	2.5486(17)	Si(2)−Sn(1)−Cl(1)	102.84(5)
Si(2)−Cl(2)	2.522(2)	Cl(2)−Si(2)−Sn(1)	73.39(5)
Si(1)-N(2)	1.839(5)	Cl(2)−Si(2)−C(15)	85.53(17)
Si(1)-N(1)	1.827(5)	C(1)-Si(1)-Sn(1)	134.01(19)
Si(2)-N(3)	1.886(5)	C(15)-Si(2)-Sn(1)	138.3(2)
Si(2)-N(4)	1.844(6)	N(1)-Si(1)-N(2)	71.6(2)
Si(1)-C(1)	1.884(6)	N(1)-Si(1)-Sn(1)	112.99(17)
Si(2)-C(15)	1.892(6)	N(2)-Si(1)-Sn(1)	110.64(17)
		N(4)-Si(2)-N(3)	70.2(2)
		N(3)-Si(2)-Sn(1)	107.67(16)
		N(4)-Si(2)-Sn(1)	108.56(18)

Table S3. Crystallographic data and structure refinement for compound **3**

Empirical formula	C49 H68 Br2 N4 O2 Si2 Sn	
Formula weight	1079.76	
Temperature	150(2) K	
Wavelength	1.54184 Å	
Crystal system	Monoclinic	
Space group	$P2_1/n$	
Unit cell dimensions	a = 13.42660(10) Å	a= 90°.
	b = 20.6091(2) Å	b= 97.1290(10)°.
	c = 18.5970(2) Å	g = 90°.
Volume	5106.20(8) $Å^3$	
Z	4	
Density (calculated)	1.405 Mg/m^3	
Absorption coefficient	6.572 mm^{-1}	
F(000)	2216	
Crystal size	0.050 x 0.030 x 0.030 mm^3	
Theta range for data collection	3.215 to 67.488°.	
Index ranges	-12<=h<=16, -24<=k<=23, -22<=l<=22	
Reflections collected	21311	
Independent reflections	9166 [R(int) = 0.0206]	
Completeness to theta = 67.488°	99.6 %	
Absorption correction	Semi-empirical from equivalents	
Max. and min. transmission	1.00000 and 0.37620	
Refinement method	Full-matrix least-squares on F^2	
Data / restraints / parameters	9166 / 0 / 557	
Goodness-of-fit on F^2	1.027	
Final R indices [I>2sigma(I)]	R1 = 0.0333, wR2 = 0.0846	
R indices (all data)	R1 = 0.0371, wR2 = 0.0876	
Extinction coefficient	n/a	
Largest diff. peak and hole	1.071 and -0.662 e.$Å^{-3}$	

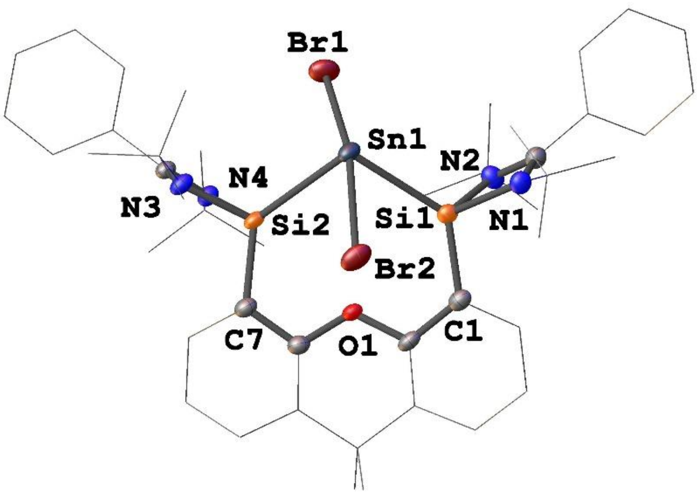

Figure **S24**. Molecular structure of compound **3**. Thermal ellipsoids are drawn at the 50% probability level. H atoms and solvent (Et_2O) molecules are omitted for clarity.

Table S4. Selected interatomic distances and angles of compound **3**.

Interatomic distances(Å)		Angles(°)	
Si(1)−Sn(1)	2.7087(7)	Si(1)−Sn(1)−Si(2)	100.81(2)
Si(2)−Sn(1)	2.6707(7)	Br(1)−Sn(1)−Br(2)	165.794(13)
Sn(1)−Br(1)	2.8651(4)	Si(1)−Sn(1)−Br(1)	97.834(18)
Sn(1)−Br(2)	2.9434(4)	Si(1)−Sn(1)−Br(2)	80.894(17)
Si(1)-N(2)	1.829(2)	Si(2)−Sn(1)−Br(1)	85.381(17)
Si(1)-N(1)	1.847(2)	Si(2)−Sn(1)−Br(2)	80.995(17)
Si(1)-C(1)	1.878(3)	C(1)−Si(1)−Sn(1)	128.22(9)
Si(2)-N(3)	1.841(2)	C(7)−Si(2)−Sn(1)	127.20(9)
Si(2)-N(4)	1.825(2)	N(2)-Si(1)-N(1)	71.39(11)
Si(2)-C(7)	1.881(3)	N(1)-Si(1)-Sn(1)	109.18(8)
		N(2)-Si(1)-Sn(1)	119.63(8)
		N(4)-Si(2)-N(3)	71.42(10)
		N(3)-Si(2)-Sn(1)	107.60(8)
		N(4)-Si(2)-Sn(1)	122.30(8)

Table S5. Crystallographic data and structure refinement for compound **4.C$_6$H$_6$**

Empirical formula	C53 H58 Fe2 N4 O9 Si2 Sn	
Formula weight	1181.60	
Temperature	150.00(10) K	
Wavelength	1.54184 Å	
Crystal system	Triclinic	
Space group	P-1	
Unit cell dimensions	a = 13.3362(3) Å	a= 100.683(2)°.
	b = 13.4474(3) Å	b= 107.806(2)°.
	c = 19.2279(5) Å	g = 96.738(2)°.
Volume	3169.90(14) Å^3	
Z	2	
Density (calculated)	1.238 Mg/m^3	
Absorption coefficient	7.494 mm^{-1}	
F(000)	1212	
Crystal size	0.02 x 0.02 x 0.01 mm^3	
Theta range for data collection	3.404 to 72.659°.	
Index ranges	-16<=h<=16, -16<=k<=13, -22<=l<=23	
Reflections collected	23962	
Independent reflections	12252 [R(int) = 0.0529]	
Completeness to theta = 67.684°	99.8 %	
Absorption correction	Semi-empirical from equivalents	
Max. and min. transmission	1.00000 and 0.51619	
Refinement method	Full-matrix least-squares on F^2	
Data / restraints / parameters	12252 / 0 / 654	
Goodness-of-fit on F^2	1.019	
Final R indices [I>2sigma(I)]	R1 = 0.0613, wR2 = 0.1642	
R indices (all data)	R1 = 0.0638, wR2 = 0.1666	
Extinction coefficient	n/a	
Largest diff. peak and hole	2.056 and -2.065 e.Å^{-3}	

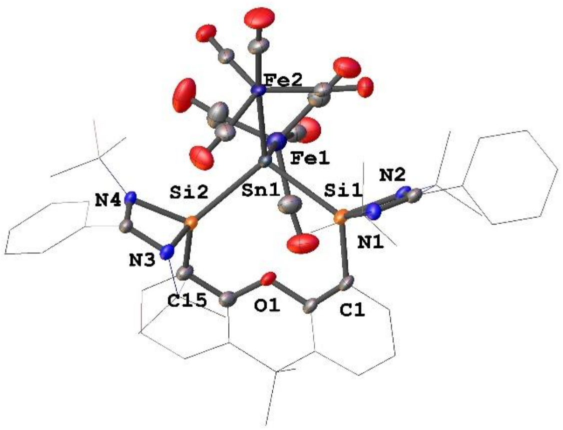

Figure **S25**. Molecular structure of compound **4**.**C_6H_6**. Thermal ellipsoids are drawn at 50% probability level. Hydrogen atoms are omitted for clarity. The strongly disordered C_6H_6 molecules are treated using Solvent Masking in Olex2.

Table S6. Selected interatomic distances and angles of compound **4**.

Interatomic distances(Å)		Angles(°)	
Si(1)−Sn(1)	2.6956(12)	Fe(2)−Sn(1)−Fe(1)	120.56(3)
Si(2)−Sn(1)	2.6797(11)	Fe(2)−Sn(1)−Si(2)	110.25(3)
Sn(1)−Fe(1)	2.6488(9)	Fe(2)−Sn(1)−Si(1)	107.47(3)
Sn(1)−Fe(2)	2.6031(7)	Fe(1)−Sn(1)−Si(2)	109.01(3)
Si(1)-C(1)	1.891(4)	Fe(1)−Sn(1)−Si(1)	106.61(3)
Si(2)-C(15)	1.867(5)	Si(2)−Sn(1)−Si(1)	101.09(4)
Si(1)-N(1)	1.833(4)	N(1)-Si(1)-N(2)	71.63(17)
Si(1)-N(2)	1.833(4)	N(4)-Si(2)-N(3)	71.70(17)
Si(2)-N(3)	1.844(4)	N(4)-Si(2)-Sn(1)	118.69(12)
Si(2)-N(4)	1.816(4)	N(3)-Si(2)-Sn(1)	129.84(13)
		N(1)-Si(1)-Sn(1)	124.36(13)
		N(2)-Si(1)-Sn(1)	118.15(12)
		C(1)-Si(1)-Sn(1)	122.33(14)
		C(15)-Si(2)-Sn(1)	116.93(16)

Table S7. Crystallographic data and structure refinement for compound **5**

Empirical formula	C45 H58 N4 O Si2 Sn

Formula weight	845.82	
Temperature	150(2) K	
Wavelength	1.54184 Å	
Crystal system	Monoclinic	
Space group	$P2_1/c$	
Unit cell dimensions	a = 19.1331(12) Å	a= 90°.
	b = 27.5473(13) Å	b= 110.956(7)°.
	c = 18.1070(12) Å	g = 90°.
Volume	8912.3(10) $Å^3$	
Z	8	
Density (calculated)	1.261 Mg/m^3	
Absorption coefficient	5.352 mm^{-1}	
F(000)	3536	
Crystal size	0.110 x 0.050 x 0.040 mm^3	
Theta range for data collection	4.140 to 67.500°.	
Index ranges	-16<=h<=22, -31<=k<=32, -21<=l<=21	
Reflections collected	36635	
Independent reflections	15972 [R(int) = 0.1643]	
Completeness to theta = 67.500°	99.4 %	
Absorption correction	Semi-empirical from equivalents	
Max. and min. transmission	1.00000 and 0.38202	
Refinement method	Full-matrix least-squares on F^2	
Data / restraints / parameters	15972 / 153 / 1032	
Goodness-of-fit on F^2	0.898	
Final R indices [I>2sigma(I)]	R1 = 0.0863, wR2 = 0.1645	
R indices (all data)	R1 = 0.2096, wR2 = 0.2208	
Extinction coefficient	n/a	
Largest diff. peak and hole	0.888 and -0.800 e.$Å^{-3}$	

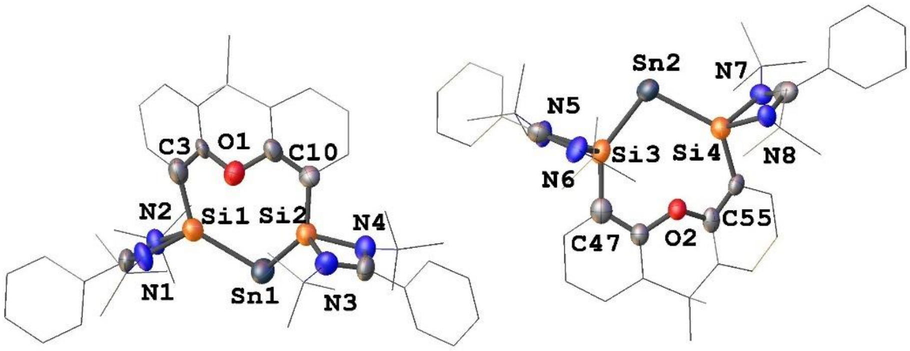

Figure S26. Molecular structure of compound **5**. Thermal ellipsoids are drawn at 50% probability level. Hydrogen atoms are omitted for clarity. (Two independent molecules ***a*** and ***b***, one of the tert-butyl groups of molecule ***a*** is disordered over two orientations with an approximate occupancy ratio of 0.75:0.25; two of the tert-butyl groups of molecule ***b*** are disordered over two orientations with an approximate occupancy ratio of 0.75:0.25 and 0.72:0.28, respectively.)

Table S8. Selected interatomic distances and angles of compound **5**.

Interatomic distances(Å)		Angles(°)	
molecule ***a*** :			
Sn(1)-Si(1)	2.520(3)	Si(1)-Sn(1)-Si(2)	99.34(10)
Sn(1)-Si(2)	2.506(3)	C(3)-Si(1)-Sn(1)	134.7(4)
Si(2)-N(4)	1.877(9)	C(10)-Si(2)-Sn(1)	133.3(4)
Si(2)-N(3)	1.904(9)	N(1)-Si(1)-N(2)	70.3(4)
Si(1)-N(1)	1.849(9)	N(4)-Si(2)-N(3)	68.7(4)
Si(1)-N(2)	1.855(8)	N(3)-Si(2)-Sn(1)	122.4(3)
Si(2)-C(10)	1.879(10)	N(4)-Si(2)-Sn(1)	110.7(3)
Si(1)-C(3)	1.901(12)	N(1)-Si(1)-Sn(1)	120.7(3)
		N(2)-Si(1)-Sn(1)	110.6(3)
molecule ***b*** :			
Sn(2)-Si(3)	2.540(3)	Si(3)-Sn(2)-Si(4)	100.10(9)
Sn(2)-Si(4)	2.535(3)	C(55)-Si(4)-Sn(2)	131.5(3)
Si(4)-N(7)	1.840(8)	C(47)-Si(3)-Sn(2)	106.6(5)
Si(4)-N(8)	1.884(8)	N(7)-Si(4)-N(8)	70.2(3)
Si(3)-N(5)	1.865(8)	N(5)-Si(3)-N(6)	70.1(4)
Si(3)-N(6)	1.870(8)	N(7)-Si(4)-Sn(2)	111.0(3)
Si(4)-C(55)	1.892(9)	N(8)-Si(4)-Sn(2)	123.9(3)
Si(3)-C(47)	1.851(11)	N(5)-Si(3)-Sn(2)	108.1(3)
		N(6)-Si(3)-Sn(2)	121.4(3)

B. DFT Calculations

Computational details. All the DFT calculations were performed with Gaussian 16 (Revision A.03) program.[4] All structures were optimized at the PEB0[5] -D3BJ[6] /Def2-SVP[7]~ma-TZVP[8] level of theory in the gas phase due to the smallest relative mean deviation (RD) of structural parameters in comparison with the experimental structure. No imaginary frequency was obtained at the same level, confirming a local minima. All presented principal interacting orbital (PIO) analyses[9] were performed by NBO 7.0 program[10] at the same level based on the optimized structure. Natural adaptive orbital (NAdO) [11] analyses were carried out by the Multiwfn program.[12] All orbitals were plotted with the help of Multiwfn and VMD programs.[13]

Gauge-Independent Atomic Orbital (GIAO) calculation. The B97-2[14]/Def2-TZVP (for all toms except Sn atom)[7]~Sapporo-DKH3-DZP-2012-diffuse (for Sn atom) [15] method is used to calculate the ^{119}Sn chemical shifts of compound **5**, where the solvent effect (solvent = benzene) is taken into accounts by the PCM model. The calculated ^{119}Sn absolute shielding constants are converted to ^{119}Sn NMR chemical shift, with $SnMe_4$ calculated at the same level as reference. **5:** ^{119}Sn NMR: $\delta_{cal.}$ = -1325.49 ppm, $\delta_{ex.}$ = -1147.24 ppm.

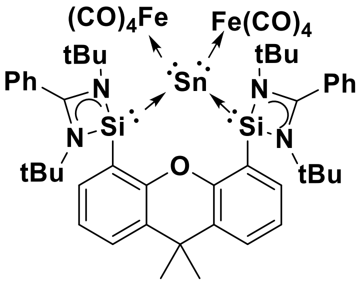

Table S9. Key distances (Å) of experimental and DFT-optimized structures of **4**.[a]

Functional	Exp.	B3PW91	PBE0	TPSS
Sn-Si1	2.696	2.647	2.679	2.681
Sn-Si2	2.68	2.635	2.665	2.584
Sn-Fe1	2.603	2.550	2.559	2.558
Sn-Fe2	2.649	2.575	2.586	2.691
∠Si1-Sn-Si2	101.1	101.0	100.5	100.5
∠Si1-Sn-Fe1	107.5	107.5	107.8	107.7
∠Fe1-Sn-Fe2	120.6	124.6	124.3	125.4
∠Fe2-Sn-Si2	109.0	108.4	108.8	108.5
RD(%)[b]	0	1.5	1.2	1.6

[a] The basis set is Def2-SVP~ma-TZVP. ma-TZVP is the abbreviation of def2-TZVP with minimal augmentation, proposed by Truhlar and co-workers.[8]

[b] $RD = \frac{\sum_{i=1}^{n}\frac{|BL(DFT)-BL(Exp.)|}{BL(Exp.)}*100\%}{n}$, BL means bond length.

Table S10. The relative electronic energy of **4 (Si_2SnFe_2)** in the two states. Here S_0 means the lowest singlet state, T_1 means the lowest triplet state.

State	**4 (Si_2SnFe_2)**
S_0	0
T_1	25.6

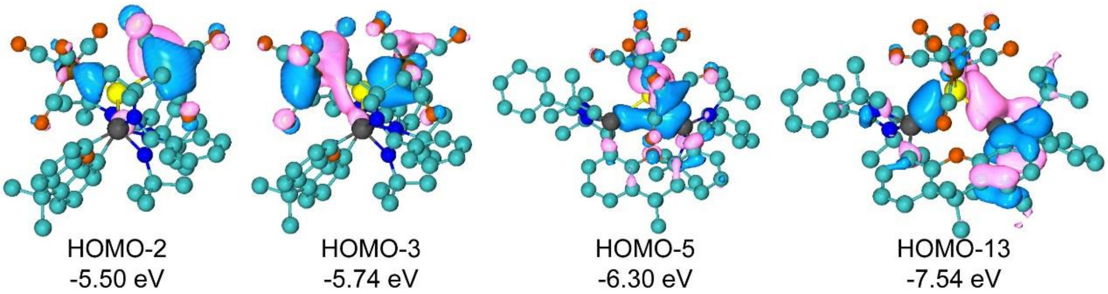

Figure S27. Frontier molecular orbital of compound **4**. Hydrogen atoms in 3D structures are omitted for clarity. The isosurfaces 0.030 au are plotted.

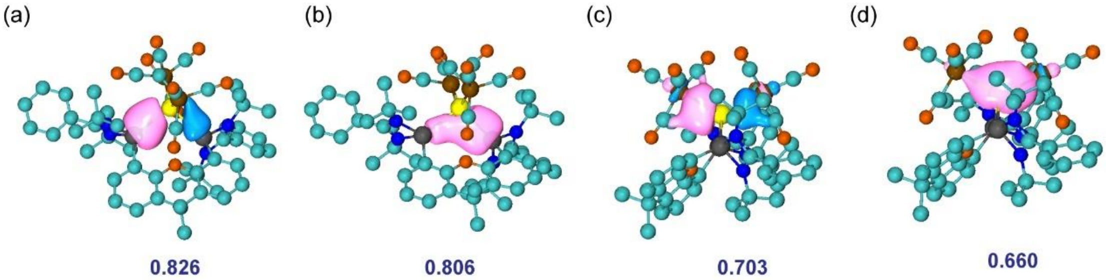

Figure S28. Four key bonding NAdOs of Sn-Si and Sn-Fe in **4**. Hydrogen atoms in 3D structures are omitted for clarity. The strength of the interaction is quantified by the eigenvalue labeled by blue. The eigenvalue sum of two Sn-Si and two Sn-Fe bonds is 3.764. The isosurface 0.050 au is plotted.

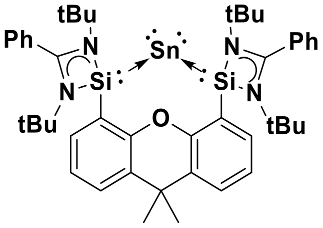

Table S11. Key distances (Å) of experimental and DFT-optimized structures of **5 (Si_2Sn)**.[a]

Functional	Exp.	TPSS
∠Sn-Si1	2.506	2.531
∠Sn-Si2	2.520	2.531
∠Si1-Sn-Si2	99.3	97.0
RD(%)[b]	0	1.3

[a] The basis set is Def2-SVP~ma-TZVP. ma-TZVP is the abbreviation of def2-TZVP with minimal.

[b] RD = $\frac{\sum_{i=1}^{n}\frac{|BL(DFT)-BL(Exp.)|}{BL(Exp.)}*100\%}{n}$,BL means bond length.

Table S12. The relative electronic energy of **5** (**Si_2Sn)** in the two states. Here S_0 means the lowest singlet state, T_1 means the lowest triplet state.

State	**5**
S_0	0
T_1	19.1

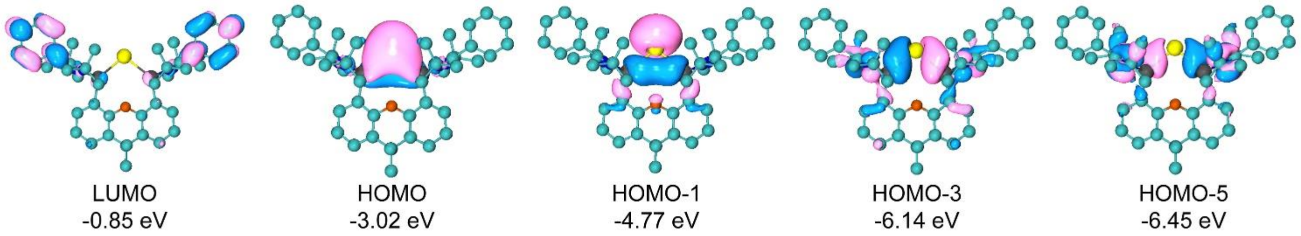

Figure S29. Frontier molecular orbital of compound **5**. Hydrogen atoms in 3D structures are omitted for clarity. The isosurfaces 0.030 au are plotted.

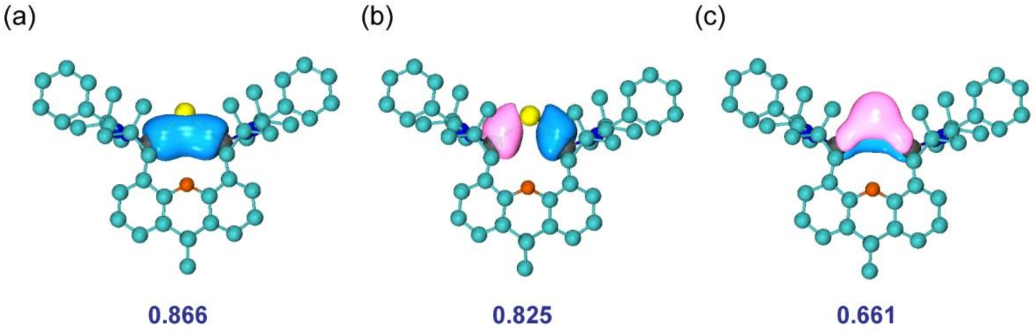

Figure S30. Three main bonding NAdOs of Sn-Si in compound **5**. Hydrogen atoms in 3D structures are omitted for clarity. The strength of the interaction is quantified by the eigenvalue labeled by blue. The eigenvalue sum of two Sn-Si is 2.570. The isosurface 0.050 au is plotted.

Cartesian Coordinates

$Si_2SnFe_2.S_0$

PBE0-D3BJ/Def2-SVP~ma-TZVP

E = -6265.493436 a.u.

Sn	0.07358100	-0.50781500	1.12851700
Fe	0.05436900	-3.06235500	0.98452600
Fe	0.57912400	0.82513400	3.28551500
Si	-2.18826000	0.41226200	0.05976500
Si	1.85685800	0.39966600	-0.65228900
O	-0.38324200	2.36919900	-0.54879400
O	2.95949200	-2.86281600	0.90149500
N	2.68337600	-0.69682800	-1.89953200
N	-3.01333400	0.02668100	-1.57500100
O	-1.09220500	-3.01500900	3.67519900
N	3.66024700	0.23549000	-0.23002300
N	-3.74256100	-0.52460800	0.37321000
O	-1.59846100	-2.77719000	-1.39626000
C	3.84920900	-0.59801200	-1.25120800
O	0.02894700	-5.95869500	0.78279600
C	0.66840400	2.96619400	-1.15338700
C	1.75399200	2.12207900	-1.42992900
C	5.11828100	-1.26862700	-1.62643600
O	2.37086000	-1.43458700	3.76366800
C	-1.50187100	3.06214700	-0.21070600
C	5.50133100	-2.46197100	-1.00719800
H	4.85641500	-2.90183600	-0.24547300
O	0.70324100	3.34968800	1.83152300
C	0.64743400	4.32874700	-1.46847400
O	1.92687800	2.21797900	5.43921200
C	-3.97270600	-0.64320700	-0.93861400
C	-2.53964300	2.24751500	0.25805300
C	2.82327700	2.66470900	-2.15060500
H	3.68196600	2.03359500	-2.39525000
C	-5.08280500	-1.40503500	-1.55986800
C	1.74907100	4.82055500	-2.17339600
H	1.77891500	5.87901700	-2.44244700
C	7.13182900	-1.31102400	-2.96403500
H	7.76867100	-0.85715500	-3.72643800
C	7.51353700	-2.50326700	-2.34836800
H	8.45176700	-2.98692500	-2.62976400
O	-1.88031800	0.54991100	4.82455500

C	-0.48060600	5.25166000	-1.00721200
C	-2.94153300	0.44011300	-2.98578300
C	2.81743100	4.00134600	-2.53674000
H	3.65633900	4.41802200	-3.09801400
C	6.69790700	-3.07547000	-1.37449900
H	6.99242400	-4.00913400	-0.89044400
C	2.26234300	-1.57146000	-3.00133300
C	5.82360600	1.41727700	0.02223600
H	5.50469800	2.14472500	-0.73948600
H	6.45768800	0.65697300	-0.45172500
H	6.44052900	1.95192200	0.75911800
C	5.93617200	-0.69570900	-2.60727800
H	5.63584900	0.23828500	-3.08695000
C	-1.60709200	4.43784200	-0.38046700
C	1.80099900	-2.91906400	0.94793900
C	-4.84798500	-2.69966600	-2.03523400
H	-3.84749100	-3.12752000	-1.94644500
C	-1.73636800	1.36586000	-3.12359400
H	-0.83431900	0.91595700	-2.69203900
H	-1.92005100	2.32602200	-2.62334000
H	-1.54180000	1.56977900	-4.18571000
C	1.65292200	-0.55980100	3.53062800
C	-0.93643500	-2.85915600	-0.43782700
C	-0.64116700	-3.00817000	2.61368900
C	-3.59509100	-0.70505900	2.74001600
H	-3.67907600	0.38738900	2.83634600
H	-2.53140100	-0.96985300	2.67613300
H	-3.97966600	-1.15703900	3.66367000
C	-3.72444000	2.86428000	0.65783800
H	-4.55449300	2.25932600	1.03161600
C	-4.35008400	-1.21633900	1.52381900
C	4.61474500	0.80862700	0.73735000
C	-6.35864000	-0.84143200	-1.66152800
H	-6.53702700	0.16898600	-1.28754900
C	0.03991800	-4.81146500	0.86958300
C	3.86799100	1.91570100	1.47237900
H	4.52887600	2.38216600	2.21646100
H	2.99626800	1.51167900	2.00839700
H	3.52458300	2.69828700	0.77958700
C	0.08187700	6.22630800	0.04259200
H	-0.70412900	6.91073900	0.39653400
H	0.89335400	6.83445700	-0.38534400
H	0.47557600	5.67149100	0.90466800
C	-5.82813900	-0.84536700	1.65650600
H	-6.43407800	-1.26282500	0.84185200
H	-5.95896300	0.24759600	1.67283400
H	-6.21830900	-1.24690100	2.60305900
C	-2.77030400	-0.77614000	-3.89832800
H	-2.57006500	-0.44269600	-4.92749900
H	-3.67700400	-1.39485500	-3.91685100
H	-1.93711300	-1.40254400	-3.55610800
C	-7.39461500	-1.56817300	-2.24252500
H	-8.38884400	-1.12363500	-2.32459300
C	-5.89180900	-3.42359300	-2.60659200
H	-5.70799700	-4.43671100	-2.97079000
C	-2.81156900	5.01643800	0.03978300
H	-2.94303200	6.09746400	-0.04975800
C	1.38807300	1.66465400	4.58621900
C	-7.16257300	-2.86009800	-2.71318900
H	-7.97753300	-3.43085500	-3.16425400
C	-1.03617200	6.02962600	-2.21090200
H	-1.43900200	5.34264400	-2.97000200
H	-0.25411500	6.64286900	-2.68182800
H	-1.84246100	6.71014900	-1.90111000
C	-4.17980300	-2.73121900	1.40892900
H	-4.53227600	-3.21177900	2.33327400
H	-3.12044700	-2.99356800	1.27180700
H	-4.76042700	-3.14306900	0.57214900
C	0.61806200	2.31357600	2.34550300
C	0.84090900	-1.14042600	-3.33875700
H	0.42159100	-1.78438200	-4.12411300
H	0.82607100	-0.10158900	-3.69961900
H	0.18788900	-1.22728300	-2.45775300
C	-0.95309900	0.60391100	4.14225400

C	2.27174500	-3.03724500	-2.56808000
H	1.61511200	-3.19063800	-1.69924100
H	3.28600500	-3.36787600	-2.30444400
H	1.91239700	-3.67173600	-3.39207700
C	5.06745200	-0.26164800	1.73078700
H	5.65882000	-1.04331800	1.23415100
H	4.20872900	-0.73740400	2.22153600
H	5.69814600	0.19822400	2.50612600
C	-3.85042500	4.25172100	0.56708700
H	-4.77073600	4.74137300	0.89233700
C	3.14258300	-1.36717800	-4.23647300
H	2.69315400	-1.89166000	-5.09272200
H	4.15381300	-1.76887000	-4.09441000
H	3.21674100	-0.29840500	-4.48973500
C	-4.19469600	1.23176900	-3.37178100
H	-4.35778000	2.06010200	-2.66530200
H	-5.09071100	0.59751900	-3.39128200
H	-4.06651500	1.65803100	-4.37813100

$Si_2SnFe_2.S_0$

TPSS-D3BJ/Def2-SVP~ma-TZVP

E = -6270.384458 a.u.

Sn	0.07647700	-0.47992900	1.15743900
Fe	0.05871000	-3.03708900	1.08272200
Fe	0.57972800	0.95178400	3.24897700
Si	-2.19899500	0.40417900	0.04894300
Si	1.86972600	0.37716800	-0.65672400
O	-0.39435700	2.33717800	-0.61137500
O	2.99848800	-2.85554800	0.98473700
N	2.69296300	-0.74565100	-1.90085200
N	-3.03432000	-0.00581500	-1.58935400
O	-1.08275700	-2.90567400	3.80964300
N	3.68562800	0.20738200	-0.23267200
N	-3.75971900	-0.53872100	0.38297500
O	-1.59387900	-2.78889800	-1.34455400
C	3.87474100	-0.64954400	-1.25276200
O	0.00971900	-5.97309800	0.96181600
C	0.67481300	2.93993100	-1.21954100
C	1.77200200	2.08878500	-1.46749000
C	5.14167400	-1.33669600	-1.62446700
O	2.38743200	-1.31365200	3.82859800
C	-1.51521300	3.05594200	-0.26006700
C	5.53075800	-2.52302800	-0.97475700
H	4.89091500	-2.94106400	-0.19435200
O	0.77932000	3.44739200	1.69430900
C	0.65049400	4.30501000	-1.55609700
O	1.90387400	2.42718900	5.40930400
C	-4.00002300	-0.68115800	-0.93841600
C	-2.55311700	2.24495000	0.23772600
C	2.85406700	2.62739400	-2.19081600
H	3.71857700	1.99279900	-2.41534200
C	-5.10912300	-1.46422700	-1.54702200
C	1.76498400	4.79223200	-2.26111400
H	1.79295200	5.84878200	-2.54802200
C	7.15166000	-1.42445100	-2.99297900
H	7.78254700	-0.99259300	-3.77650600
C	7.53841500	-2.60867000	-2.34680900
H	8.47332400	-3.10475900	-2.62729400
O	-1.93427800	0.78117600	4.77822000
C	-0.49537600	5.23677000	-1.12930900
C	-2.98186000	0.40929300	-3.01574200
C	2.84642000	3.96520700	-2.60154100
H	3.69142600	4.37672000	-3.16264200
C	6.72707900	-3.15462600	-1.34252200
H	7.02405900	-4.07900100	-0.83696400
C	2.24571500	-1.66027700	-2.97873600
C	5.87374200	1.39170600	-0.02497900
H	5.54904800	2.10029500	-0.80607500
H	6.49570700	0.60841500	-0.48509000
H	6.50200400	1.94239100	0.69544100
C	5.95582100	-0.79074700	-2.63683900
H	5.65150400	0.13336800	-3.13754300
C	-1.61143300	4.43747200	-0.44723100
C	1.82563400	-2.89810800	1.03556000
C	-4.86038900	-2.76702800	-2.02120200

H	-3.85193900	-3.18112000	-1.93818000
C	-1.76643300	1.33694200	-3.17089500
H	-0.86142500	0.88197300	-2.74294100
H	-1.94533500	2.30063000	-2.66908200
H	-1.58402400	1.53500600	-4.23973600
C	1.66090100	-0.44114200	3.54772600
C	-0.93301400	-2.85154700	-0.36673400
C	-0.63593900	-2.92793600	2.73102700
C	-3.59173700	-0.68769200	2.76378700
H	-3.73908000	0.40083500	2.85609400
H	-2.51239000	-0.88912200	2.66777400
H	-3.92924100	-1.16435300	3.69683700
C	-3.73365700	2.87932000	0.65534900
H	-4.56215300	2.28317800	1.05223400
C	-4.34554800	-1.24709800	1.55481500
C	4.65884700	0.80652600	0.72342900
C	-6.40313600	-0.91718000	-1.63912900
H	-6.59219500	0.09427300	-1.26720100
C	0.03038300	-4.80979000	1.01698600
C	3.91126300	1.94154900	1.43717900
H	4.57690500	2.42115900	2.17354800
H	3.03292200	1.55016900	1.97801300
H	3.57256900	2.70913500	0.72117000
C	0.06251700	6.29187400	-0.13698800
H	-0.73858200	6.98081800	0.18254000
H	0.85737800	6.89058100	-0.61464000
H	0.47737300	5.79312200	0.75207400
C	-5.84533400	-0.91760700	1.68660200
H	-6.43837300	-1.37314700	0.87836800
H	-6.00823900	0.17424800	1.67911700
H	-6.21738700	-1.31450500	2.64624500
C	-2.82834400	-0.82206000	-3.93096300
H	-2.64243000	-0.49028800	-4.96708200
H	-3.74207900	-1.43632000	-3.92889200
H	-1.98889100	-1.44856900	-3.59578500
C	-7.44021000	-1.66662500	-2.20893000
H	-8.44382100	-1.23574200	-2.28302500
C	-5.90577300	-3.51318000	-2.58054100
H	-5.71099700	-4.52825500	-2.94111000
C	-2.81150500	5.03239600	-0.00871300
H	-2.93865400	6.11531900	-0.11183900
C	1.37257900	1.84100700	4.55464000
C	-7.19334700	-2.96542300	-2.67790300
H	-8.00656900	-3.55159200	-3.11821200
C	-1.08126000	5.94125800	-2.38127700
H	-1.48085500	5.19966400	-3.09372800
H	-0.30708200	6.53640900	-2.89494700
H	-1.89802900	6.62609500	-2.09677800
C	-4.12410300	-2.76715900	1.44744500
H	-4.47607000	-3.25367000	2.37284900
H	-3.05229500	-2.99378300	1.32354100
H	-4.68395800	-3.19575700	0.60065500
C	0.66227200	2.41255700	2.23626000
C	0.82381000	-1.19886900	-3.32529400
H	0.37560900	-1.86941100	-4.07594100
H	0.83919400	-0.17452800	-3.73363500
H	0.18149800	-1.22176400	-2.42838300
C	-0.98612100	0.78653300	4.09780700
C	2.22697600	-3.11801000	-2.48213300
H	1.57021600	-3.22044800	-1.60271200
H	3.24069700	-3.45276000	-2.20840500
H	1.85089100	-3.77809000	-3.28284700
C	5.11493100	-0.25235000	1.74529300
H	5.69176400	-1.05289600	1.25461500
H	4.25307100	-0.70402400	2.25772200
H	5.76328700	0.22443600	2.50053500
C	-3.85075700	4.27492500	0.55133500
H	-4.76344700	4.77601400	0.88921400
C	3.13676200	-1.51959400	-4.22926500
H	2.67141900	-2.06762200	-5.06651100
H	4.14078200	-1.94010200	-4.06690100
H	3.23507100	-0.45914900	-4.51959700
C	-4.25180200	1.20719500	-3.37995200
H	-4.39433200	2.04176000	-2.67195700

H	-5.14912400	0.56875300	-3.36880600
H	-4.14577500	1.62498400	-4.39617800

$Si_2SnFe_2.S_0$

B3PW91-D3BJ/Def2-SVP~ma-TZVP

E = -6268.666702 a.u.

Sn	0.07778800	-0.47798100	1.12563400
Fe	0.07111000	-3.02629200	1.02799600
Fe	0.57818700	0.90307100	3.24008100
Si	-2.17359100	0.39747600	0.07364700
Si	1.83771800	0.38420400	-0.65346600
O	-0.39619900	2.34364600	-0.56546300
O	2.97817100	-2.80584700	0.93890800
N	2.65664800	-0.73213200	-1.88248900
N	-2.98880400	0.00080900	-1.55944000
O	-1.04587300	-2.94210700	3.73521900
N	3.63716300	0.22065600	-0.22677200
N	-3.70990300	-0.56034100	0.38959500
O	-1.59066700	-2.77312300	-1.35495500
C	3.82548300	-0.62562400	-1.23846400
O	0.05492700	-5.92988200	0.86001200
C	0.65107200	2.93610000	-1.18550700
C	1.73813300	2.09100800	-1.45215800
C	5.09399600	-1.29172500	-1.61446800
O	2.32758400	-1.37667400	3.80068900
C	-1.51475500	3.03924200	-0.22491000
C	5.50019400	-2.46694000	-0.97450200
H	4.86960100	-2.89741000	-0.19672600
O	0.72253700	3.40332700	1.74132100
C	0.62252700	4.29365300	-1.52210500
O	1.95409100	2.33496400	5.35648600
C	-3.94166300	-0.68045200	-0.92259600
C	-2.54237600	2.22547100	0.26781100
C	2.80727800	2.62523700	-2.18095500
H	3.66678900	1.99230100	-2.41551500
C	-5.04819800	-1.44692700	-1.53948700
C	1.72316300	4.77943500	-2.23506400
H	1.74874900	5.83338000	-2.52101900
C	7.09082700	-1.33563400	-2.97901700
H	7.71180200	-0.88989100	-3.75919900
C	7.49672700	-2.51060000	-2.34249800
H	8.43768000	-2.98786200	-2.62603500
O	-1.90052000	0.72656000	4.77005700
C	-0.51497600	5.21728200	-1.08094100
C	-2.93042400	0.42495300	-2.96771900
C	2.79638600	3.95775000	-2.58541900
H	3.63413300	4.36987300	-3.15196700
C	6.70075900	-3.07325600	-1.34506200
H	7.01393700	-3.99278700	-0.84575400
C	2.22415600	-1.63443700	-2.95707900
C	5.81189800	1.38737000	-0.00115400
H	5.50313400	2.08852600	-0.79085000
H	6.44129800	0.60513500	-0.44408300
H	6.42920200	1.94311600	0.71964300
C	5.89086400	-0.72840100	-2.61923100
H	5.57054700	0.19112400	-3.11261400
C	-1.62815000	4.41138700	-0.41768200
C	1.81806100	-2.86952500	0.98689700
C	-4.81055900	-2.74079400	-2.01725500
H	-3.80799300	-3.16284800	-1.93389600
C	-1.71689500	1.34117200	-3.11308100
H	-0.81845800	0.88497800	-2.68233000
H	-1.89129900	2.30297200	-2.61426300
H	-1.52334000	1.54188800	-4.17574800
C	1.62665900	-0.49538400	3.53261800
C	-0.92562600	-2.84079600	-0.39459500
C	-0.60894500	-2.94873200	2.66554600
C	-3.55166000	-0.73187700	2.75319600
H	-3.68415600	0.35497400	2.85015300
H	-2.47771600	-0.94613400	2.67706800
H	-3.90482900	-1.20291900	3.67941400
C	-3.72819900	2.84036600	0.66993300
H	-4.55030100	2.23567500	1.06009300
C	-4.29302100	-1.27358400	1.54011000
C	4.59258800	0.81332600	0.72749100

C	-6.32719000	-0.88681600	-1.63164000
H	-6.50560300	0.12132800	-1.25260600
C	0.06229300	-4.77928900	0.93408000
C	3.84763400	1.94663700	1.42594200
H	4.50256500	2.42951800	2.16450700
H	2.96948300	1.55937100	1.96272700
H	3.51341100	2.71044400	0.70901700
C	0.04287300	6.23702000	-0.06975300
H	-0.74947300	6.92221100	0.26769100
H	0.84164600	6.84174500	-0.52511900
H	0.45164100	5.71670200	0.80612800
C	-5.78468400	-0.95382400	1.67091000
H	-6.37426600	-1.40015900	0.85982100
H	-5.95382200	0.13376500	1.67595700
H	-6.16055000	-1.36034500	2.62113900
C	-2.78297200	-0.79053700	-3.88780900
H	-2.58354700	-0.45732500	-4.91714100
H	-3.69871100	-1.39538000	-3.90194300
H	-1.95745600	-1.42904500	-3.55104600
C	-7.36498100	-1.61696800	-2.20772100
H	-8.36159700	-1.17634800	-2.28256200
C	-5.85596300	-3.46853500	-2.58347000
H	-5.67095400	-4.48074000	-2.94978600
C	-2.83250900	4.99066900	0.00647100
H	-2.97206100	6.06889500	-0.10042300
C	1.40315000	1.76602700	4.51841600
C	-7.13061300	-2.90874300	-2.68195000
H	-7.94629300	-3.48200800	-3.12870700
C	-1.09215400	5.94305100	-2.30987000
H	-1.49128100	5.22109600	-3.03768300
H	-0.32201000	6.54731200	-2.81111400
H	-1.90568900	6.62330200	-2.01890600
C	-4.06875500	-2.78198800	1.41880100
H	-4.40932500	-3.28093000	2.33773000
H	-3.00064100	-3.00502100	1.28550600
H	-4.62968600	-3.20656000	0.57527400
C	0.62823800	2.37145900	2.26703100
C	0.80337000	-1.19755400	-3.29663300
H	0.36531200	-1.86475100	-4.05118300
H	0.79885100	-0.17292400	-3.69511300
H	0.16066500	-1.24076900	-2.40537000
C	-0.96652000	0.73479400	4.09090000
C	2.22675800	-3.08684900	-2.47704300
H	1.57168400	-3.20731100	-1.60265800
H	3.24019900	-3.41081500	-2.20303400
H	1.86368200	-3.74727400	-3.27865400
C	5.02628200	-0.23684100	1.75216500
H	5.60704300	-1.03935100	1.27729300
H	4.15751400	-0.68700000	2.24760700
H	5.65938400	0.23299600	2.51950300
C	-3.86206100	4.22717200	0.55820100
H	-4.78179400	4.71719700	0.88492400
C	3.10335800	-1.47265000	-4.20111200
H	2.64551900	-2.01330000	-5.04279100
H	4.10961700	-1.88289200	-4.04993400
H	3.19067300	-0.41174600	-4.48092700
C	-4.18484600	1.23026600	-3.32668600
H	-4.32999200	2.05078600	-2.60763200
H	-5.08422900	0.60080600	-3.33617800
H	-4.07195200	1.66644600	-4.33067900

$Si_2SnFe_2.T_1$

PBE0-D3BJ/Def2-SVP~ma-TZVP

E = -2833.217037 a.u.

Sn	0.06396200	-0.68494100	0.89898800
Fe	0.03534000	-3.18242000	0.26913300
Fe	0.88861400	-0.10156700	3.70777900
Si	-2.09939200	0.50491900	0.00110100
Si	1.68846300	0.52021400	-0.77411000
O	-0.12886700	2.61001100	0.26138000
O	2.93836300	-2.93943000	0.21205100
N	2.62137200	-0.30914900	-2.14525700
N	-3.10429900	0.37575800	-1.57009100
O	-1.06288800	-3.65529500	2.93648900
N	3.48193800	0.33340500	-0.29001100

N	-3.65749400	-0.42814800	0.34057200
O	-1.70050900	-2.48301600	-1.96590000
C	3.75756500	-0.27919200	-1.43711600
O	0.02031300	-5.97825900	-0.51008200
C	0.74225700	3.19781200	-0.60401500
C	1.63097500	2.34597000	-1.26140000
C	5.07771700	-0.79657000	-1.87366400
O	2.45306900	-2.61689900	3.41198900
C	-1.36426400	3.18257600	0.38167500
C	5.49601200	-2.08017700	-1.50971300
H	4.84392100	-2.70110200	-0.89394600
O	-0.58183900	2.44632400	3.25816500
C	0.70139400	4.58494800	-0.78240800
O	2.55999300	0.90713400	5.93964300
C	-4.01763900	-0.36426000	-0.94897100
C	-2.43885900	2.30035300	0.47245900
C	2.49701500	2.93325200	-2.19496300
H	3.20381800	2.30314900	-2.74195400
C	-5.18554000	-1.02710200	-1.57626000
C	1.57066500	5.12196300	-1.73288900
H	1.57150500	6.19663900	-1.92262300
C	7.14732300	-0.45917800	-3.07780600
H	7.79138600	0.17712100	-3.68872600
C	7.56363700	-1.74106400	-2.71822000
H	8.53672600	-2.11179700	-3.04846400
O	-1.28230700	-1.32198600	5.31795200
C	-0.21589300	5.38759800	0.13886300
C	-3.06608500	0.96688100	-2.91087100
C	2.45533500	4.30289400	-2.44060800
H	3.12470600	4.74614800	-3.18131700
C	6.73780800	-2.54707500	-1.93715900
H	7.06016500	-3.55113700	-1.65284900
C	2.26809700	-0.99256500	-3.39633100
C	5.42945600	1.74459300	0.25915500
H	4.97853100	2.55755800	-0.33040700
H	6.15585900	1.21030700	-0.36773700
H	5.97973200	2.19361100	1.09932400
C	5.90684300	0.01252000	-2.65942900
H	5.57701300	1.01473000	-2.94161600
C	-1.49102000	4.57101800	0.32103500
C	1.77799500	-3.01520300	0.23860900
C	-5.00739000	-2.24785500	-2.23666100
H	-4.01147800	-2.69428500	-2.27419500
C	-1.86062700	1.90749600	-2.92801100
H	-0.94697400	1.38318300	-2.61250600
H	-2.01615300	2.76286400	-2.25479100
H	-1.69659200	2.29723200	-3.94256700
C	1.84711700	-1.64460200	3.40874800
C	-1.00167200	-2.70850700	-1.05852400
C	-0.63407700	-3.44623800	1.88345100
C	-3.32201900	-0.88416800	2.66685600
H	-3.31091600	0.19840200	2.86991100
H	-2.28888500	-1.22065900	2.50632000
H	-3.69115300	-1.40012700	3.56330900
C	-3.71346300	2.86044000	0.61661600
H	-4.58403000	2.20298600	0.69439600
C	-4.19926800	-1.21077600	1.46183700
C	4.34515700	0.80787800	0.79822800
C	-6.45385500	-0.43997000	-1.52047800
H	-6.58710800	0.51646600	-1.01026000
C	0.02631400	-4.87079300	-0.19589000
C	3.44366500	1.59548000	1.74662100
H	4.02149800	1.95278500	2.61038400
H	2.62509400	0.96443000	2.13056700
H	2.99964100	2.46802800	1.24384800
C	0.48050900	5.51049600	1.51066300
H	-0.16169200	6.06150800	2.21443300
H	1.43608800	6.04710000	1.40697800
H	0.68100100	4.52089500	1.94472100
C	-5.63630000	-0.78247300	1.76714900
H	-6.32423500	-1.04917400	0.95406000
H	-5.69270400	0.30317400	1.94123400
H	-5.98381100	-1.29075400	2.67857500
C	-2.90272000	-0.11335100	-3.98129300

H	-2.75678500	0.35581300	-4.96600400
H	-3.79453400	-0.75226000	-4.04024300
H	-2.04086400	-0.75447400	-3.75722100
C	-7.53951600	-1.07146600	-2.12171200
H	-8.52784200	-0.60849300	-2.07883400
C	-6.09974900	-2.87643800	-2.82998700
H	-5.95921300	-3.83255200	-3.33898500
C	-2.78201200	5.08882200	0.44594900
H	-2.94025700	6.16798800	0.40930500
C	1.90749700	0.50017400	5.08833100
C	-7.36394200	-2.29112000	-2.77461800
H	-8.21727600	-2.78727500	-3.24267100
C	-0.50305200	6.77975500	-0.41173400
H	-0.99962600	6.73506100	-1.39255800
H	0.42730700	7.35574200	-0.51746700
H	-1.14508900	7.34541500	0.27815000
C	-4.13515500	-2.70889700	1.16214000
H	-4.40959800	-3.28254300	2.05948400
H	-3.11640900	-3.00040500	0.86784000
H	-4.82868700	-2.98656800	0.35608100
C	0.00883500	1.46594200	3.35112700
C	0.84806700	-0.53940400	-3.72216900
H	0.48554800	-1.04066900	-4.63069700
H	0.81164700	0.54780900	-3.88894000
H	0.16193400	-0.79745400	-2.90153200
C	-0.44376000	-0.86696400	4.68393700
C	2.29439100	-2.50970200	-3.21123200
H	1.60235100	-2.81547900	-2.41282700
H	3.30327800	-2.86204400	-2.95465400
H	1.98957300	-3.00518100	-4.14523200
C	4.96904800	-0.37490200	1.53955200
H	5.67239500	-0.92274400	0.89739300
H	4.19541500	-1.07817400	1.87529900
H	5.52240100	-0.01518900	2.41976300
C	-3.88090700	4.24368700	0.62006100
H	-4.87859300	4.67415100	0.73233100
C	3.19891800	-0.57366800	-4.53607700
H	2.81535300	-0.97751400	-5.48471500
H	4.21835400	-0.95662700	-4.39865500
H	3.24164300	0.52279500	-4.62595500
C	-4.33184300	1.78919200	-3.16669700
H	-4.47905700	2.52727300	-2.36346900
H	-5.22398000	1.15165400	-3.23360500
H	-4.23544600	2.33140600	-4.11940900

$Si_2Sn.S_0$

PBE0-D3BJ/Def2-SVP~ma-TZVP

E = -2833.247451 a.u.

Sn	-0.00018500	-1.44674900	-1.07278000
Si	1.89528600	0.08702100	-0.39260500
Si	-1.89531400	0.08724500	-0.39208100
O	0.00038400	2.18377300	0.43261900
N	-3.50741000	-0.57831000	-1.08996100
N	3.05548600	-0.49530800	1.00329400
N	3.50719400	-0.57919800	-1.09025100
N	-3.05547100	-0.49572500	1.00357400
C	2.20483700	1.95789000	-0.32609700
C	1.16026900	2.79831300	0.05524100
C	0.00046700	6.40648500	0.08536600
H	0.88253000	6.93920000	0.46864700
H	-0.88124500	6.93929000	0.46932900
H	0.00004500	6.47770400	-1.01239300
C	3.52231100	3.96683000	-0.69618600
H	4.46310700	4.43554900	-0.99490100
C	-3.97465600	-0.93652300	-2.43296500
C	3.40940000	2.57783000	-0.68612800
H	4.26082000	1.95947300	-0.98274700
C	1.22759400	4.19382100	0.05921500
C	-3.93213100	-0.95077400	0.12401000
C	3.93197100	-0.95102400	0.12389800
C	2.43689400	4.76896000	-0.33763700
H	2.54249800	5.85498700	-0.36166000
C	-1.22689400	4.19394300	0.06013500
C	-1.15969900	2.79842400	0.05604800
C	0.00057400	4.95548900	0.55298400

C	-3.52217400	3.96721300	-0.69363400
H	-4.46314400	4.43603800	-0.99163000
C	-5.01139300	-3.15123300	0.57388800
H	-4.03651200	-3.62440000	0.43523400
C	-3.40935500	2.57820700	-0.68382000
H	-4.26101000	1.95995000	-0.97997100
C	2.92127800	-0.62641400	2.45032800
C	3.97433100	-0.93823400	-2.43307700
C	-2.20458600	1.95813100	-0.32469100
C	-7.49219500	-1.92542000	0.92942100
H	-8.46228900	-1.44359300	1.07083200
C	5.47968500	-0.71205900	-2.58778500
H	5.75545900	0.31099000	-2.28858300
H	5.76017300	-0.84299300	-3.64363500
H	6.07165500	-1.42147900	-1.99488000
C	-2.43642400	4.76921800	-0.33580800
H	-2.54193900	5.85525600	-0.35969200
C	-3.62372300	-2.39621100	-2.73576700
H	-2.54038400	-2.55906800	-2.62508400
H	-3.91045200	-2.65419100	-3.76685300
H	-4.15424800	-3.07876800	-2.05565700
C	4.26203100	-0.50480400	3.17455600
H	4.78816200	0.41279300	2.87043400
H	4.91905100	-1.36393700	2.98516500
H	4.08280400	-0.45265300	4.25909600
C	5.13232400	-1.76568100	0.43181300
C	3.23233300	-0.02205600	-3.40763300
H	2.14258000	-0.14352000	-3.30266700
H	3.51270600	-0.26835400	-4.44238900
H	3.47906000	1.03370300	-3.22144500
C	-3.23260900	-0.01984900	-3.40701900
H	-3.47918700	1.03582800	-3.22015600
H	-3.51308200	-0.26547700	-4.44190700
H	-2.14286800	-0.14152400	-3.30219100
C	5.01093800	-3.15137500	0.57504600
H	4.03597200	-3.62448000	0.43675600
C	-5.13258000	-1.76542900	0.43153300
C	6.37823200	-1.15532700	0.61006000
H	6.46789100	-0.07234800	0.50128700
C	2.01063800	0.52270400	2.88731500
H	2.48375600	1.49547500	2.68747900
H	1.78893600	0.44986300	3.96211400
H	1.05897100	0.48775600	2.33554600
C	-6.37839000	-1.15501000	0.61022900
H	-6.46790700	-0.07195100	0.50214200
C	-5.47999400	-0.71010000	-2.58744200
H	-6.07199700	-1.41977200	-1.99487000
H	-5.76057300	-0.84044200	-3.64334200
H	-5.75562900	0.31282100	-2.28767700
C	-6.13043200	-3.91910900	0.89024900
H	-6.03097100	-5.00155900	0.99814300
C	-7.37048200	-3.30838200	1.06986500
H	-8.24606300	-3.91154700	1.32072800
C	0.00115800	4.91566700	2.09570100
H	0.00123300	3.87868600	2.46195200
H	-0.89398800	5.41977000	2.49178000
H	0.89666200	5.41966300	2.49110300
C	-2.92119700	-0.62767600	2.45052700
C	6.12987900	-3.91921700	0.89183500
H	6.03027000	-5.00158500	1.00041200
C	2.25131100	-1.96524200	2.77600300
H	2.04557100	-2.04205900	3.85508800
H	2.90141300	-2.80590400	2.49149500
H	1.30582300	-2.05743000	2.21934700
C	3.62321800	-2.39805700	-2.73502100
H	4.15365800	-3.08027800	-2.05450900
H	3.90991800	-2.65668400	-3.76595300
H	2.53986000	-2.56072000	-2.62423600
C	7.37002600	-3.30855600	1.07099400
H	8.24553100	-3.91169100	1.32219700
C	7.49194000	-1.92569900	0.92967700
H	8.46211500	-1.44393100	1.07073000
C	-2.01016600	0.52092100	2.88808700
H	-1.05853000	0.48593500	2.33625700

H	-1.78841600	0.44742200	3.96282900
H	-2.48296600	1.49395900	2.68879100
C	-2.25160500	-1.96689000	2.77539300
H	-1.30621900	-2.05909600	2.21856100
H	-2.90199600	-2.80718200	2.49047500
H	-2.04578100	-2.04437400	3.85441800
C	-4.26185800	-0.50606300	3.17492400
H	-4.78770100	0.41188700	2.87138400
H	-4.08253500	-0.45460100	4.25948200
H	-4.91917800	-1.36487200	2.98507500

$Si_2Sn.T_1$

PBE0-D3BJ/Def2-SVP~ma-TZVP

E = -2833.217037 a.u.

Sn	-0.02352400	-1.22064400	-1.26237500
Si	1.96372700	0.19083300	-0.36339000
Si	-2.09306100	0.18352400	-0.41234000
O	0.01884300	2.22462800	0.45989000
N	-3.67972200	-0.35845900	-1.08663100
N	2.94724800	-0.65149300	0.98515800
N	3.56244500	-0.47117300	-1.05878200
N	-2.91692000	-0.63905200	0.95032000
C	2.27877700	2.03289000	-0.13165400
C	1.20088500	2.85267700	0.20976900
C	0.07515300	6.46771200	0.32496400
H	0.93422000	6.97626300	0.78539500
H	-0.82387900	6.99525000	0.67315900
H	0.14494500	6.58219900	-0.76691700
C	3.63396400	4.04089500	-0.28089300
H	4.59735200	4.51896700	-0.47229300
C	-3.94208000	-0.84793300	-2.45015500
C	3.51405000	2.65607300	-0.35753600
H	4.38274400	2.04568400	-0.61791700
C	1.28056200	4.24643100	0.29311500
C	-3.97802600	-1.01752600	0.12543200
C	3.86461600	-1.04897500	0.10967400
C	2.52223500	4.82703100	0.03002800
H	2.63316000	5.91168400	0.07689700
C	-1.17140700	4.27095500	0.14186900
C	-1.12821900	2.87537400	0.08269000
C	0.03141400	4.99937400	0.73393800
C	-3.44962800	4.12697500	-0.68374500
H	-4.37063900	4.62789600	-0.99095300
C	-4.74874500	-3.12843200	1.17409600
H	-3.71915100	-3.34301400	1.46350100
C	-3.37269800	2.73589900	-0.70271600
H	-4.22714700	2.13143700	-1.01821300
C	2.70665300	-0.95600400	2.39546500
C	4.08853400	-0.68150300	-2.41276300
C	-2.19482300	2.07574300	-0.32696500
C	-7.36121900	-2.64891700	0.33879100
H	-8.39084100	-2.43970900	0.03439900
C	5.59287800	-0.40992100	-2.46622800
H	5.82431200	0.59172600	-2.07273200
H	5.93753200	-0.45241600	-3.51004800
H	6.16538400	-1.15257300	-1.89480300
C	-2.35689900	4.88667000	-0.26656800
H	-2.43840100	5.97478400	-0.24669600
C	-3.57094800	-2.32671100	-2.59700900
H	-2.50444700	-2.48837200	-2.37264800
H	-3.75814700	-2.67335400	-3.62533300
H	-4.16283000	-2.94801300	-1.90952400
C	3.99353900	-0.87817700	3.21644200
H	4.49665600	0.08794100	3.05937900
H	4.69505600	-1.68488000	2.96547200
H	3.74875100	-0.96721700	4.28523300
C	4.97662500	-1.99196900	0.37608800
C	3.36370400	0.32243700	-3.30960000
H	2.27502600	0.15359900	-3.28409600
H	3.70020300	0.21114200	-4.35027200
H	3.56083100	1.35597500	-2.98886100
C	-3.08273800	0.00517200	-3.38720100
H	-3.34635500	1.07008200	-3.29810700
H	-3.23296800	-0.30346900	-4.43201600
H	-2.00811400	-0.10147400	-3.16343700

C	4.74845100	-3.37084300	0.32689400
H	3.75502400	-3.74675100	0.07171800
C	-5.01480300	-1.95024300	0.41616600
C	6.24844500	-1.50715500	0.69841100
H	6.42090300	-0.42957000	0.74104500
C	1.72911200	0.11329500	2.88144700
H	2.17964800	1.11517600	2.82216400
H	1.43072700	-0.07939000	3.92167000
H	0.81710300	0.11010300	2.26492800
C	-6.36594200	-1.74064700	0.01710800
H	-6.62886700	-0.81154100	-0.48811200
C	-5.40245500	-0.64508500	-2.86927100
H	-6.07133500	-1.36247600	-2.38020500
H	-5.49891300	-0.78655300	-3.95666100
H	-5.73652600	0.37476700	-2.62394000
C	-5.75377500	-4.02875500	1.48616900
H	-5.50367700	-4.93315400	2.04841100
C	-7.07478700	-3.80871900	1.07231500
H	-7.86413000	-4.52056900	1.32191400
C	-0.06551000	4.89949800	2.27131300
H	-0.10091900	3.84948100	2.59693200
H	-0.98019800	5.39730300	2.62789300
H	0.80668900	5.37785600	2.74341200
C	-2.86069600	-0.48421600	2.40485100
C	5.78888300	-4.25712600	0.59764200
H	5.60719200	-5.33327900	0.55563900
C	2.06500600	-2.34017000	2.52385200
H	1.76974800	-2.53152100	3.56644700
H	2.76637800	-3.12994000	2.21872900
H	1.16820600	-2.40645600	1.88902600
C	3.78223900	-2.10409000	-2.88783300
H	4.32080700	-2.85019400	-2.28626500
H	4.09160300	-2.22981100	-3.93640000
H	2.70220700	-2.30647400	-2.81741700
C	7.05497500	-3.77221300	0.92150200
H	7.86836000	-4.46903000	1.13605300
C	7.28319700	-2.39699700	0.97177000
H	8.27391600	-2.01444900	1.22682300
C	-2.13453800	0.82972300	2.70961800
H	-1.15695300	0.87095200	2.20490800
H	-1.95564500	0.92817200	3.79109900
H	-2.72452500	1.69451300	2.37401400
C	-2.05255800	-1.63159200	3.02545500
H	-1.05649400	-1.68460600	2.56070700
H	-2.54975500	-2.59839700	2.87393700
H	-1.92504600	-1.48188100	4.10935400
C	-4.25456500	-0.40988900	3.03165300
H	-4.85608500	0.36929600	2.54056300
H	-4.15886600	-0.15390700	4.09817800
H	-4.80006300	-1.35847200	2.95392100

C. TD-DFT Calculations

Computational details. Time dependent density functional theory (TD-DFT) were performed to calculate UV-vis spectra, under TD-B3LYP-D3BJ/Def2-SVP~ma-TZVP level including solvent effect in a SMD continuum model (solvent= toluene),[16] due to the good agreement with experimental UV-vis spectra.

Results of UV-vis calculation：According to the TD-DFT calculation, the observed peak ($\lambda_{ex.}$=674 nm, $\lambda_{TD\text{-}DFT.}$=672 nm, Figure S31) is mainly assigned to the S_1 state (Table S13), which corresponds to the π-π* excitation from HOMO to LUMO orbital (Table S13).

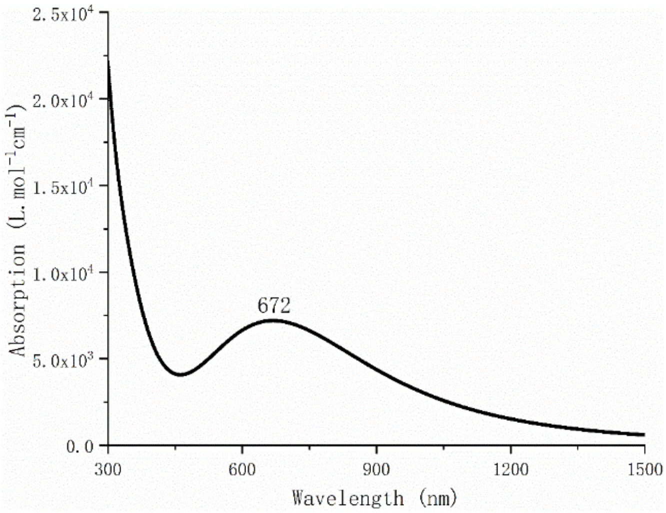

Figure S31. TD-DFT absorption spectra for **5**.

Table S13. Main contributions of individual transitions to the peak ($\lambda_{TD\text{-}DFT}$ = 672 nm).

Transition	Contribution
S_1	54.2%
S_7	15.2%
S_2	13.5%
S_3	10.6%

Table S14. Oscillator strengths and transitions in different states of the compound **5** by TD-DFT calculations.

state	Main transition	Wavelength (nm)	Oscillator strength
S_1	HOMO→LUMO (98.8%)	726.5	0.154
S_7	HOMO→LUMO+6 (89.3%)<br>HOMO→LUMO (9.2%)	567.8	0.055
S_2	HOMO→LUMO+1 (82.3%)<br>HOMO→LUMO+2 (14.1%)	685.8	0.036
S_3	HOMO→LUMO+2 (73.1%)<br>HOMO→LUMO+1 (17.2%)<br>HOMO→LUMO+6 (7.8%)	669.2	0.028

References

[1] Y. Wang, A. Kostenko, S. Yao, M. Driess, *J. Am. Chem. Soc.* **2017**, *139*, 13499-13506.
[2] J. Gladysz, W. Tam, *J. Chem. Phys.* **1978**, *43*, 2279-2280.
[3] G. M. Sheldrick, SHELX-97 Program for Crystal Structure Determination, *University of Göttingen, Germany,* **1997**.
[4] M. Frisch, G. Trucks, H. Schlegel, G. Scuseria, M. Robb, J. Cheeseman, G. Scalmani, V. Barone, G. Petersson, H. Nakatsuji in Gaussian, Inc. Wallingford, CT, **2016**.
[5] C. Adamo, V. Barone, *J. Chem. Phys.* **1999**, *110*, 6158-6170.
[6] S. Grimme, S. Ehrlich, L. Goerigk, *J. Comput. Chem.* **2011**, *32*, 1456-1465.
[7] F. Weigend, R. Ahlrichs, *PCCP* **2005**, *7*, 3297-3305.
[8] a) J. Zheng, X. Xu, D. G. Truhlar, *Theor. Chem. Acc.* **2011**, *128*, 295-305; b) E. Papajak, J. Zheng, X. Xu, H. R. Leverentz, D. G. Truhlar, *J. Chem. Theory Comput.* **2011**, *7*, 3027-3034.
[9] a) J. X. Zhang, F. K. Sheong, Z. Lin, *WIREs Comput Mol Sci.* **2020**, *10*, e1469; b) J. X. Zhang, F. K. Sheong, Z. Lin, *Chem. Eur. J.* **2018**, *24*, 9639-9650.
[10] E. D. Glendening, C. R. Landis, F. Weinhold, *J. Comput. Chem.* **2019**, *40*, 2234-2241.
[11] J. L. Casals-Sainz, A. Fernández-Alarcón, E. Francisco, A. Costales, A. Martin Pendas, *J. Phys. Chem. A* **2019**, *124*, 339-352.
[12] T. Lu, F. Chen, *J. Comput. Chem.* **2012**, *33*, 580-592.
[13] W. Humphrey, A. Dalke, K. Schulten, *J. Mol. Graph. Model.* **1996**, *14*, 33-38.
[14] P. J. Wilson, T. J. Bradley, D. J. Tozer, *J. Chem. Phys.* **2001**, *115*, 9233-9242.
[15] a) T. Noro, M. Sekiya, T. Koga, *Theor. Chem. Acc.* **2012**, *131*, 1-8; b) B. P. Pritchard, D. Altarawy, B. Didier, T. D. Gibson, T. L. Windus, *J. Chem. Inf. Model.* **2019**, *59*, 4814-4820.
[16] A. V. Marenich, C. J. Cramer, D. G. Truhlar, *J. Phys. Chem. B* **2009**, *113*, 6378-6396.
